# Miktoarm Star Polymers: Branched Architectures in Drug Delivery

**DOI:** 10.3390/pharmaceutics12090827

**Published:** 2020-08-30

**Authors:** Victor Lotocki, Ashok Kakkar

**Affiliations:** Department of Chemistry, McGill University, 801 Sherbrooke Street West, Montreal, QC H3A 0B8, Canada; victor.lotocki@mail.mcgill.ca

**Keywords:** drug delivery, macromolecules, miktoarm polymers, heteroarm star polymers, synthesis, self-assembly, soft nanoparticles, micelles, nanoformulations

## Abstract

Delivering active pharmaceutical agents to disease sites using soft polymeric nanoparticles continues to be a topical area of research. It is becoming increasingly evident that the composition of amphiphilic macromolecules plays a significant role in developing efficient nanoformulations. Branched architectures with asymmetric polymeric arms emanating from a central core junction have provided a pivotal venue to tailor their key parameters. The build-up of miktoarm stars offers vast polymer arm tunability, aiding in the development of macromolecules with adjustable properties, and allows facile inclusion of endogenous stimulus-responsive entities. Miktoarm star-based micelles have been demonstrated to exhibit denser coronae, very low critical micelle concentrations, high drug loading contents, and sustained drug release profiles. With significant advances in chemical methodologies, synthetic articulation of miktoarm polymer architecture, and determination of their structure-property relationships, are now becoming streamlined. This is helping advance their implementation into formulating efficient therapeutic interventions. This review brings into focus the important discoveries in the syntheses of miktoarm stars of varied compositions, their aqueous self-assembly, and contributions their formulations are making in advancing the field of drug delivery.

## 1. Introduction

A majority of active pharmaceutical agents fail to provide expected relief upon administration as 90% of drugs in the discovery pipeline have very poor water solubility and low bioavailability [1,2,3]. These and other related shortcomings, including untargeted accumulation and systemic toxicity, have necessitated the development of nanocarriers for efficient therapeutic interventions. In particular, much progress has been made in the development of soft polymeric nanoparticles over the last 30 years, and it has contributed significantly to enhancing drug solubility, stability, long circulation times, and targeting specific locations in the body [4]. To achieve a free energy minimum, amphiphilic polymers with distinct hydrophilic and hydrophobic blocks self-assemble in an aqueous medium, into a range of supramolecular structures, including micelles and polymersomes [5,6]. Such nanoparticles can accumulate at disease sites using the enhanced permeation and retention (EPR) effect [7,8,9], resulting from the porous leaky vasculature typical of unhealthy cancerous tissue and its deficient lymphatic drainage [10,11,12].

Micelles are supramolecular assemblies that constitute the majority of polymeric nanocarriers, and are characterized by their distinct core-corona build-up. The outer hydrophilic corona contributes to the solubility and stability of micellar structures in aqueous media [9], and in the majority of the nanoparticles, it is almost exclusively composed of, or based on, poly(ethylene glycol) (PEG). PEG owes its ubiquity to its ability to confer aqueous solubility, stealth, and compatibility with biological systems by avoiding immunogenic response and premature elimination [4]. Using their hydrophobic cores, micelles can load a variety of cargo, including small hydrophobic drugs and biomolecules. A key parameter which is intrinsic to the function of polymeric micelles as drug delivery vehicles is the critical micelle concentration (CMC). At concentrations below their CMC, amphiphilic polymers are disordered. At concentrations at or above the CMC, the continued addition of amphiphiles leads to the formation of their self-assemblies (micelles). Upon administration and introduction into the aqueous biological environment (typically blood), micelles are subject to immense dilution, and low CMCs are required for long circulation times [4,13]. Micellar drug delivery formulations based on diblock or graft copolymers generally have CMC in a range of 10^−4^–10^−7^ M, but are generally more common in the lower end of this range [4,14,15,16,17]. Another parameter of importance is the micelle size, which is typically expected to be below 200 nm. Given the vasculature and tissue pore sizes, such small diameters can significantly improve micellar circulation and biological distribution, and aid in disease site targeting, due to the EPR effect [10,11,12].

**Figure 1:**
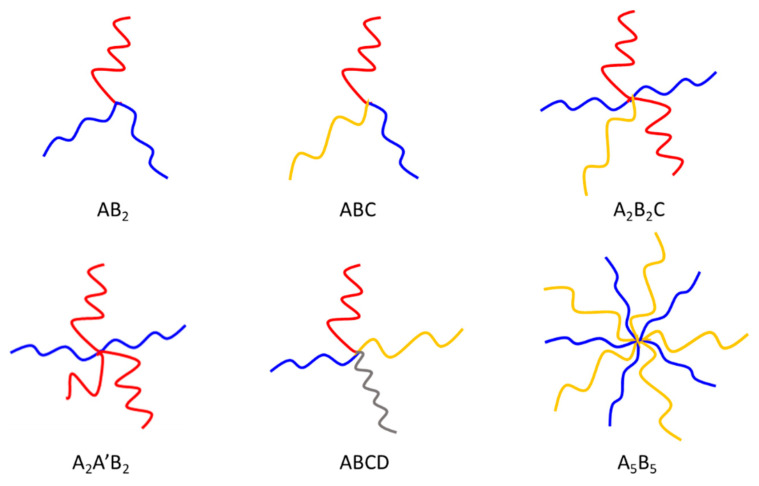
Miktoarm polymer architectures of varied compositions.

One of the more fascinating and advantageous approaches to improving polymer-based drug delivery has come from adjusting the architecture of polymeric backbones. Miktoarm polymers (sometimes known as heteroarm star polymers) are a class of star polymers with asymmetric branching in which at least three branching strands originate from a shared core [18,19]. Their compositions differ from slight variations in molecular weight, to having completely different repeating units and chemical configurations. Due to their asymmetry, miktoarm variants are categorized in the form: A_x_B_y_C_z_, where A, B, and C are examples of polymeric chains, and the subscript denotes their number (Figure 1). The branching architectures of miktoarm polymers have contributed distinct properties to their aqueous self-assemblies, compared to those from their linear diblock copolymer counterparts, including very low CMCs, smaller sizes, and most importantly the ability to encapsulate large amounts of drug molecules [20,21,22,23,24,25]. In addition, the tunability offered by having multiple branching segments has led to the synthesis of a variety of micelle structures that incorporate polymeric arms with stimulus-responsive units and biological targeting moieties. These are in addition to conferring aqueous solubility and maintaining micelle stability. Table 1 below provides a brief summary, in chronological order of their discovery, of the different types of miktoarm polymeric architectures and their assemblies, used for the delivery of a variety of pharmaceutics [21,25,26]. It shows the diversity in their composition, and the potential of targeting these formulations to desired sites through the introduction of various stimuli.

## 2. Synthetic Approaches to Miktoarm Star Polymers

**Figure 2:**
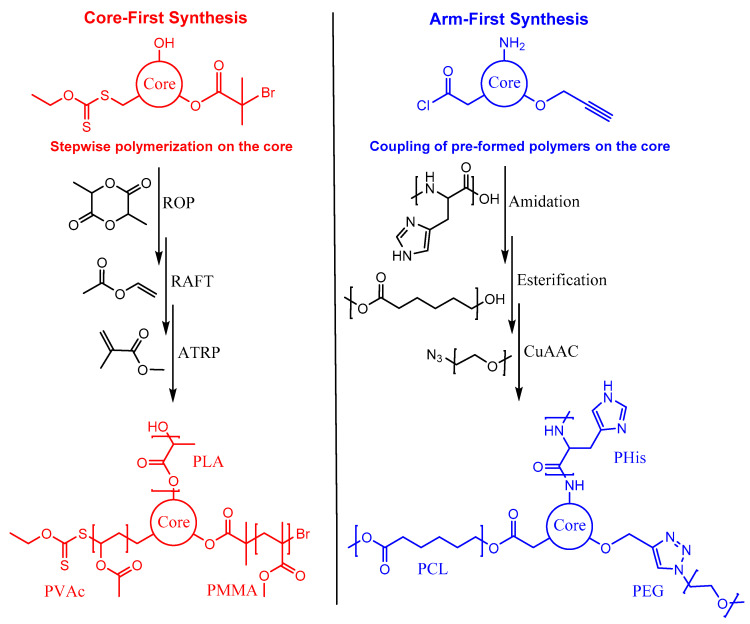
Examples of core-first synthesis (**left**) and arm-first synthesis (**right**) with examples of functionalities, monomers, and polymers.

Given the asymmetric nature of miktoarm stars, their construction requires careful selection of high yield methodologies for the build-up of individual arms on a branched core. Akin to the divergent and convergent synthetic methodologies pioneered by Tomalia and Fréchet for the synthesis of hyperbranched dendrimers [79,80], the construction of branched miktoarm polymers can be mostly broken down to two methods: arm-first and core-first (Figure 2). These involve the independent synthesis of separate polymeric arms, before attachment to a core molecule, or polymerization initiated on a hetero-multifunctional core, respectively [21]. Depending on the necessary reaction conditions, cores may be functionalized separately, or have certain moieties blocked, in order to initiate polymerization from specific locations. Alternatively, in arm-first approaches, pre-synthesized polymers with conjugating end moieties can be coupled to these cores using esterification/amidation-based coupling, or, more recently, “click” chemistry [81]. Both arm-first and core-first methods present their own advantages depending on whether a miktoarm polymer is densely branched, requires precise arm lengths, needs to be synthesized with ease, etc. However, it has become more common recently to assemble miktoarm stars using a mixture of the arm- and core-first approaches. Such an approach can best accommodate varied conditions required for the construction of desired branched architectures. For example, considering that one of the most widely studied applications of miktoarm polymers is in biology, and especially as soft nanoparticle-based drug delivery, PEG has become an increasingly featured component of miktoarm polymers. Thus, it is often much simpler to purchase PEG of a desired molecular weight, modify it to contain a reactive end group, and couple it to cores that have been used for the initiation of hydrophobic polymer polymerization [32,33,34,35,36,37,40,41].

### 2.1. Chlorosilane Based Synthesis

**Scheme 1:**
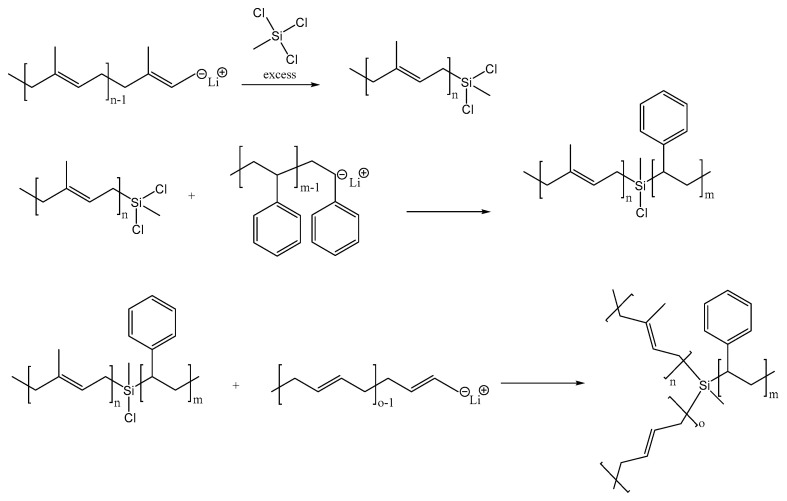
Development of an ABC miktoarm polymer based on polyisoprene (PI), polystyrene (PS), and polybutadiene (PB), using the chlorosilane method.

Hadjichristidis and coworkers were the first to prepare polymers with asymmetric branching based on an arm-first method using chlorosilane cores [82,83,84,85,86,87]. Dubbed “miktoarm” after the Greek word μικτός (miktos) or “mixed”, these polymers had a variety of branches emanating from a single core. More specifically, the first example of such a polymer was a construct made of polyisoprene (PI), polystyrene (PS), and polybutadiene (PB). Due to the increasing reactivity of the polymer anion termini in the order of PB > PI > PS, and the steric hindrance that follows the opposite trend, the miktoarm synthetic methodology was designed such that PI (with intermediate reactivity) would first be linked to a large excess of a chlorosilane core. Then, relying on the inability of the second PS arm to undergo complete reaction with PI-linked chlorosilane, PS was attached second, followed by excess PB, which would undergo complete exchange with the remaining Cl on the chlorosilane core (Scheme 1) [88].

While this PB-PI-PS star, as described above, is mostly recognized as the first synthesized miktoarm polymer, a few years prior to this, Pennisi and Fetters had reported A_2_A′ polymers based on PS and PB arms with differing molecular weights, synthesized using a chlorosilane core-based approach [86]. Then in 1990, an AB_2_-type miktoarm polymer was prepared by the single grafting of PS end-capped with chlorosilane, onto the middle of a PI chain [89].

Iatrou and Hadjichristidis further took advantage of relative polymer chain reactivities to synthesize a variety of branched polymers. Using a tetrachlorosilane core, they were able to sequentially add anionic PS, poly(4-methyl styrene) (P4MS), PI, and PB lithium salts, following each step with a titration, and confirming the ABCD miktoarm star end product via size exclusion chromatography (SEC). By swapping the middle components in the sequential addition with PS and PB, respectively, an A_2_B_2_ miktoarm polymer was prepared [90].

Due to the possible manipulations of chlorosilane cores in developing miktoarm architectures, more methodologies soon followed. While many of these included alterations of polymer addition onto trichlorosilane or tetrachlorosilane cores [91,92,93,94,95,96,97,98], one of the more interesting examples was the synthesis of an A_8_B_8_ (A = PS, B = PI) miktoarm polymer based on a core with 16 active chlorosilane bonds (called Si-Cl_16_ for simplicity). This core was synthesized by transforming a tetravinylsilane initial core with methyldichlorosilane using vinylmagnesium bromide. PS, which was made into a living polymer chain with BuLi, was carefully reacted with Si-Cl_16_ to produce PS_8_-(Si-Cl_8_). After product confirmation by SEC, the remaining chlorosilane bonds were used to link living PI chains to yield a PS_8_PI_8_ miktoarm polymer [99]. Hadjichristidis and coworkers later used a different chlorosilane linker containing 6 Si-Cl bonds, 1,2-bis(trichlorosilyl)ethane, as the core for an AB_5_ (A = PS, B = poly(2-methyl-1,3-pentadiene) (P2MP)) miktoarm polymer. To avoid multiple PS conjugations, a living PS chain was slowly added dropwise to the core in solution, after which P2MP was used to completely react with the remaining chlorosilane bonds [100]. Chlorosilane based synthesis has found great success over the years, and it paved the way for other miktoarm polymers. This methodology is well suited for the introduction of non-polar polymeric chains, and has largely been used with chains that have living anionic ends.

### 2.2. Core-First Synthesis

**Scheme 2:**
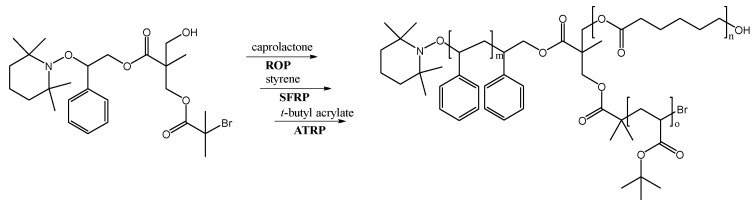
Core-first synthesis of an ABC miktoarm polymer (A = PCL, B = PS, C = PtBA) from a multifunctional core, using varied polymerization techniques.

This methodology, as the name suggests, involves initiating polymerization from a core, and incorporates standard polymerization techniques that do not involve coupling or conjugation of separately synthesized polymeric chains. It includes methods, such as atom transfer radical polymerization (ATRP), reversible addition-fragmentation chain transfer (RAFT), anionic/cationic polymerization, and ring-opening polymerization (ROP). A notable early example demonstrating the flexibility and power of core-first methods was the synthesis of a trifunctional initiator molecule, with a (2,2,6,6-Tetramethylpiperidin-1-yl)oxyl (TEMPO) group for the stable free radical polymerization (SFRP) of styrene, a bromoisobutyrate for ATRP of *t*-butyl acrylate, and a free OH for ROP of caprolactone (Scheme 2) [101]. Each of these polymerization methods could be applied to a wide variety of different monomers. For example, substituting caprolactone with ethylene oxide for ROP would yield a PEG chain in place of PCL, thus making an amphiphilic miktoarm polymer suitable for aqueous self-assembly.

Core-first synthesis has also been used to prepare amphiphilic miktoarm polymers for drug delivery. A series of A_2_B, A_2_B_2_, AB_3_, and A_3_B (A = polycaprolactone (PCL), B = poly(oligo(ethylene glycol) monomethyl ether methacrylate) (POEGMA)) miktoarm polymers, were synthesized from pentaerythritol and 1,1,1-tris(hydroxymethyl)ethane, tetrafunctional and trifunctional cores bearing 4 or 3 hydroxyls, respectively. Protecting/deprotecting strategies were used to functionalize these cores with α-bromoisobutyryl bromide, an ATRP initiator. Unfunctionalized OH positions were then used to initiate the polymerization of caprolactone, after which ATRP of OEGMA was carried out at the remaining Br activated positions. The miktoarm polymers were found to have no significant cytotoxicities, could be self-assembled into micelles, and were able to efficiently encapsulate and deliver doxorubicin (DOX) to cells. Interestingly, the most hydrophobic PCL_3_POEGMA derived micelles had the lowest CMC, the highest DOX encapsulation efficiency, and the best therapeutic efficiency for DOX release [70].

**Scheme 3:**
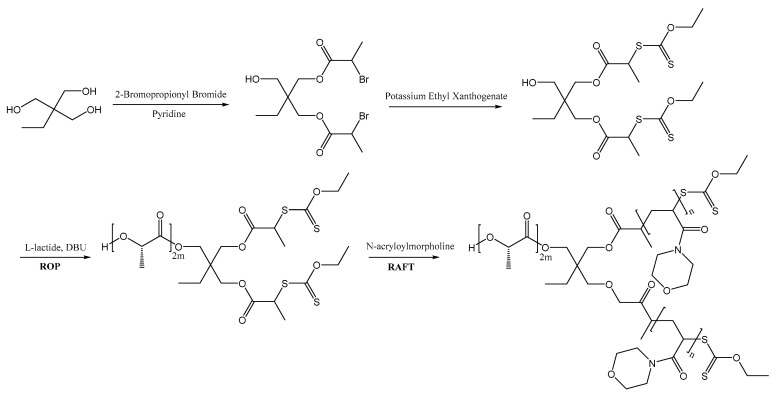
Core-first synthesis of an AB_2_ miktoarm polymer (A = PLLA, B = PNAM) from a multifunctional core using ring-opening polymerization (ROP) and reversible addition-fragmentation chain transfer (RAFT).

Core-first methods are compatible with a variety of polymerization reactions. An AB_2_ (A = poly(L-lactide) (PLLA), B = poly(N-acryloylmorpholine) (PNAM)) miktoarm polymer was prepared using a 1,1,1-tris(hydroxymethyl)ethane core, as in the above study. However, 2-bromopropionyl bromide and potassium ethyl xanthogenate were sequentially coupled onto two of the core’s hydroxy groups, to yield a multifunctional core containing two RAFT initiating groups and a free hydroxy group. The latter was employed to initiate the ROP of L-lactide, and subsequently the two RAFT initiating ends were used for the polymerization of N-acryloylmorpholine to give the final AB_2_ polymer (Scheme 3) [72]. Since most amphiphilic polymers use PEG to confer aqueous solubility, PNAM is an interesting substitute noted for its low cytotoxicity, and promising properties, including its high aqueous solubility and low cytotoxicity [102,103].

In another study, a “macro-core” was generated by conducting the anionic ROP of glycidol from methoxy PEG, leading to a highly branched polyglycerol (PG). The ROP of caprolactone was then performed using the PG terminal hydroxy ends as initiators to yield an AB_10_ (A = PEG, B = PCL) miktoarm polymer, with good loading efficiency and sustained release of prednisone acetate, an adrenocortical hormone drug [37]. The methodology used in this procedure can be argued to be of a mixed type, and in fact, most miktoarm polymer syntheses in drug delivery do use a combination of core-first and arm-first methods. Due to the complexity in preparing hetero-multifunctional cores for polymerization initiation without side reactions, mixed methodologies are often more practical.

### 2.3. Arm-First Synthesis

Arm-first syntheses generally comprise methodologies in which polymer chains are individually prepared, and then attached to a single core. In fact, the chlorosilane-based miktoarm polymers described above belong to the arm-first methodology. This approach is advantageous due to the great control over the properties of individual chains, and the freedom to use reaction conditions that would normally be incompatible in the presence of other polymers. The most important aspect of these syntheses is the necessity to use a coupling or conjugation reaction to link a completed polymer chain to the core. This can generally be achieved using (i) a condensation reaction between a terminal alcohol/amine and acid; and (ii) “click” coupling chemistry. Due to the unlikelihood of complete reaction between polymer terminal ends, which can generally be associated with steric factors, condensations are conducted using catalysts. For example, Steglich famously described an esterification procedure that uses a combination of dicyclohexylcarbodiimide (DCC) and 4-dimethylaminopyridine (DMAP) as a coupling reagent and catalyst, respectively [104].

Depending on the reaction conditions, necessary workup, and catalyst solubility, a variety of combinations of coupling reagents have been employed, including, but not limited, to: 1-Ethyl-3-(3-dimethylaminopropyl)carbodiimide (EDC), *N,N*′-Diisopropylcarbodiimide (DIC), DCC, *N*-Hydroxysuccinimide (NHS), 1,4-Dimethylpyridinium p-toluenesulfonate (DPTS), and DMAP. For example, an AB_2_ miktoarm polymer was prepared with two PEG segments conjugated to a benzyl core using click chemistry, and niacin was coupled to the benzyl alcohol on the core using EDC/DMAP coupling chemistry. The niacin conjugated structure was seen to localize within cellular lipid droplets (LD), and inhibit the activity of the LD-localized enzyme, DGAT2 [41]. Another AB_2_ (A = PEG, B = PLLA) miktoarm polymer was synthesized by first activating an acid-terminated PEG with NHS/DCC, to couple it with the free amine of serinol, thereby providing a PEG-core structure where the core has two free hydroxy groups available for ROP. L-Lactide was subsequently polymerized using these terminal alcohols as initiators to give the final miktoarm polymer [34].

Click coupling is the other widely used form of chemical conjugation applied in developing miktoarm polymer architectures. The term “click chemistry”, while having older roots, is now widely used to refer mainly to the copper-catalyzed alkyne-azide cycloaddition (CuAAC) coupling reaction, first demonstrated by Sharpless [105]. Due to this reaction’s atom economy, tendency to only form the 1,4 isomer, complete or near-complete reaction progression, tolerance of other functional groups, and capacity to be adapted into an immense variety of reaction conditions, it has found significant usage in polymer coupling, in general [81]. An example of its use in miktoarm polymer synthesis can be found in one of the first miktoarm polymers used in drug delivery. An α-methoxy-ω-epoxy poly(ethylene glycol) was exposed to sodium azide in the presence of ammonium chloride in order to produce a methoxy PEG-core structure with primary azide and hydroxy functional groups. A propargyl alcohol-initiated PCL chain was then combined with this PEG, and reacted in the presence of 1,8-diazabicyclo[5.4.0]undec-7-ene (DBU) and CuI, to “click” the two polymers together. The remaining hydroxy functional group was used for the ROP of 2-Ethoxy-2-oxo-1,3,2-dioxaphospholane (EEP), thus forming an ABC (A = PEG, B = PCL, C = polyphosphoester (PPE)) miktoarm star [106].

**Scheme 4:**
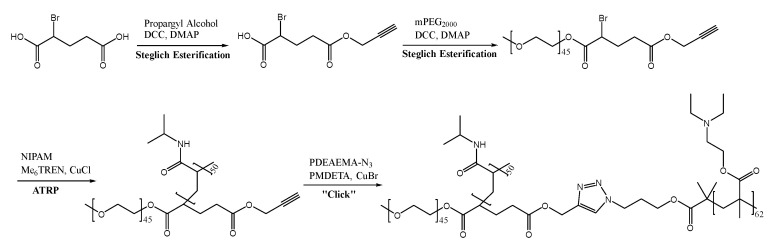
Synthesis of an ABC (A = PEG, B = PNIPAM, C = PDEAEMA) miktoarm star polymer using sequential arm-first (Steglich esterification, CuAAC “click” coupling) and core-first (core-initiated ATRP—atom transfer radical polymerization) methods.

Most researchers rarely limit themselves to one type of conjugation or even only arm-first syntheses. Instead, arm-first and core-first methods are typically combined to attain the desired miktoarm star polymer. For example, both Steglich esterification and CuAAC coupling were combined with a core-first methodology. Propargyl alcohol and mPEG_2000_ were coupled to 2-bromoglutaric acid (BGA), in this sequence, through DCC/DMAP catalyzed Steglich esterifications to yield the desired PEG_2000_(-alkynyl)-Br macroinitiator. The latter was used for the ATRP of *N*-isopropylacrylamide (NIPAM) with tris(2-(dimethylamino)ethyl)amine (Me_6_TREN) and CuCl. Finally, azido-terminated poly(2-(diethylamino)ethyl methacrylate (PDEAEMA), prepared earlier through ATRP, was “clicked” to the alkynyl group of the PEG-PNIPAM diblock, using CuAAC coupling with *N*,*N*,*N*′,*N*″,*N*″-Pentamethyldiethylenetriamine (PMDETA) and CuBr, giving the ABC (A = PEG, B = PNIPAM, C = PDEAEMA) miktoarm star polymer (Scheme 4) [107].

### 2.4. In-Out Synthesis

Synthesis of miktoarm polymers using “in-out” methodologies does not strictly fall into either core-first or arm-first methods. In-out syntheses typically involve the preparation of polymeric macroinitiators, and subsequently crosslinking them using small molecules, such as those containing divinyl functionalities. Miktoarm polymers prepared in this manner form densely core-crosslinked star (CCS) A_x_B_y_ architectures, comprised of two different arm variants. The first known example of such a miktoarm polymer involved the synthesis of vinylbenzyl terminated PS and PI by coupling living anionic PS and PI with *p*-chloromethylstyrene. These macroinitiators were then copolymerized in benzene with n-BuLi as an initiator to yield A_x_B_y_ (A = PS, B = PI) miktoarm stars. The latter were reported to undergo microphase separation, with individual domains being smaller than those observed with the analogous diblock copolymers [108]. While this study did not use polymeric arms compatible with drug delivery, such microdomain sizes suggest potentially beneficial micelle sizes and properties for such architectures.

**Scheme 5:**
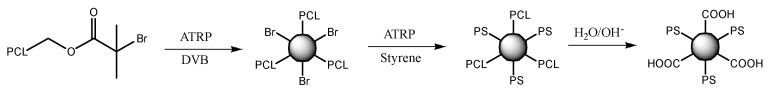
In-out synthesis of A_x_B_y_ (A = PCL, B = PS) core-crosslinked star (CCS) polymers and their subsequent alkaline hydrolysis.

An example of a typical in-out methodology involved modification of PCL with 2-bromoisobutyryl bromide to form PCL-Br, a macroinitiator for ATRP, which was subsequently core-crosslinked with divinylbenzene (DVB) to give a PCL star polymer with ATRP-active ends. The latter were then used for the polymerization of styrene to yield an A_x_B_y_ (A = PCL, B = PS) CCS polymer. The PCL chains in this miktoarm star were able to undergo biodegradation through hydrolysis in alkaline conditions (Scheme 5) [109]. Using a poly(butyl acrylate) (PBA) macroinitiator for ATRP, Matyjaszewski’s group was similarly able to demonstrate ATRP-enabled core crosslinking using DVB. This was followed by using the resulting Br-capped ends to initiate polymerization of PS. A biodegradable miktoarm polymer was similarly synthesized by exchanging a few components from their earlier miktoarm polymer. Poly(methyl methacrylate) (PMMA) polymerization was initiated using 2-bromoisobutyrate and CuBr/2,2′-bipyridine (bpy) as a catalyst. The resulting chain was then core crosslinked using bis(2-methacryloyloxyethyl) disulfide, a divinyl molecule containing a degradable disulfide linkage. Subsequent ATRP of BA led to the formation of a biodegradable A_x_B_y_ (A = PBA, B = PMMA) miktoarm polymer. Reduction, and the resulting degradation of the miktoarm polymer, was triggered by its incubation in 0.08 M Bu_3_P/THF solution, and complete cleavage was seen in 40 h [110]. While not carried out under biological conditions, a comparison can be made to the reductive power of intracellular glutathione (GSH), present at roughly 10 mM concentrations, which can cleave disulfide functional groups [111,112,113].

A similar study approached the idea of biodegradable CCS polymers, which included arm-degradable A_x_, partially arm-degradable A_x_B_y_, and core-degradable B_y_ (A = PCL, B = PS/PMMA). Beginning with a bifunctional core, 2-hydroxyethyl 2′-methyl-2′-bromopropionate, which had both ROP and ATRP active ends, arm-degradable polymers were synthesized by the sequential ROP of CL, and core-crosslinking using DVB. This resulted in a PCL-CCS polymer, which could be degraded through hydrolysis. A non-degradable analogue was synthesized by using the initiator for the ATRP of MMA/St first, followed by DVB crosslinking. Most interestingly, however, was the capacity to make partially arm-degradable A_x_B_y_ polymers using this approach [114]. This opens up the possibility of synthesizing miktoarm polymer-based drug delivery systems with tuned biological responses that can be adjusted to provide a good sustained release of drug cargo.

**Figure 3:**
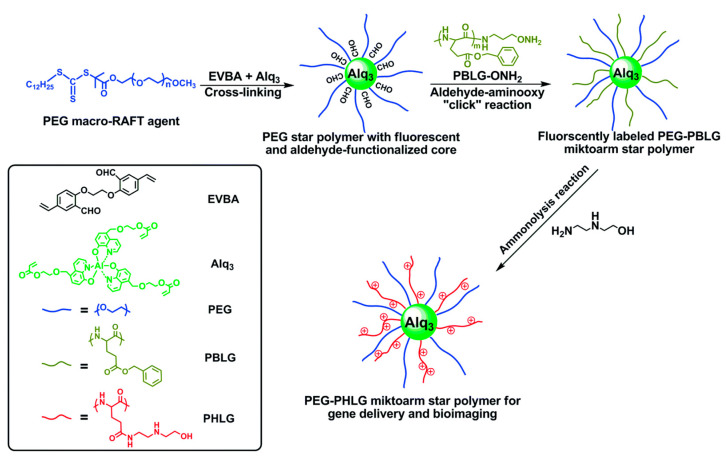
Schematic representation of PEG-PHLG CCS polymer synthesis using an in-out methodology. Reprinted with permission from reference [61]. Copyright 2016 Royal Society of Chemistry.

To our knowledge, there is one example of a CCS miktoarm star that has been evaluated for drug delivery. Trithiocarbonate-terminated polyethylene glycol (PEG-DMPA), a PEG-based RAFT macroinitiator, was synthesized from α-methoxy-ω-hydroxy PEG. It was core crosslinked with 6,6′-(ethane-1,2-diylbis(oxy))bis(3-vinylbenzaldehyde) (EVBA), a divinyl linker with pendant aldehyde functionalities, in the presence of aluminum tris(8-hydroxyquinoline) (Alq_3_), a fluorescent crosslinker, to yield a CCS with PEG arms, short aldehyde pendants, and a crosslinked fluorescent core. The aldehyde groups were used to couple with aminooxy end-functionalized poly(γ-benzyl-L-glutamate) (PBLG), a polypeptide, through the aldehyde-aminooxy click reaction. After an aminolysis step with β-hydroxyethylenediamine, the PBLG arms were converted to poly(β-hydroxyethylenediamine-L-glutamate) (PHLG), which is cationic at physiological pH (Figure 3). The resulting charged miktoarm polymers were found to be biocompatible and were able to bind siRNA through electrostatic interactions. Due to their surface charge, the miktoarm polymers were easily taken up by cancer cells where they could deliver their siRNA cargo. At the same time, the fluorescent miktoarm cores provided efficient tracking [61].

### 2.5. Miktoarm Polymer Characterization

Owing to their branched architecture, the characterization of miktoarm polymers is important to establish their compositions, and it requires careful consideration and interpretation of data. Almost universally, the characterization of miktoarm star build-up is obtained through a combination of NMR spectroscopy and GPC chromatography. Despite its prowess in characterizing polymer homoarms, MALDI-TOF has not been successfully employed for the characterization of miktoarm polymers [53,67,115].

Many of the earlier miktoarm polymers, generally synthesized using chlorosilane-based methodologies, had ABC type structures, which were simple to elucidate. For example, as discussed in Section 2.1, the construction of the first miktoarm polymer required separate anionic polymerization of PI, PS, and PB arms—each of which were characterized using GPC. The stepwise conjugation of the polymers with living anionic ends, onto a chlorosilane core, was then followed by GPC, and a downwards shift in retention time, upon addition of each arm, was observed. The inclusion of each arm was further determined using ^1^H NMR, and through this combination, miktoarm polymer’s overall composition could be accurately calculated [88]. Due to its reliability and simplicity, this characterization methodology has been applied in recent ABC type miktoarm polymers, which have been used for applications in drug delivery [42,45]. For example, the synthesis of a PEG-PNBM-PNIPAM miktoarm polymer, which involved sequential ATRP of NBM, and CuAAC of alkynyl-PNIPAM onto a PEG(-Br)-N_3_ macroinitiator, was followed stepwise, using GPC, ^1^H NMR, as well as FT-IR [66].

Characterization of miktoarm polymers with AB_n_ composition typically involves characterization using a careful comparison of ^1^H NMR peak integrations, together with the analysis of their GPC chromatograms. For example, in an AB_2_ polymer where B segments are added by click chemistry, the relative integrations of B-derived protons become double, compared to those from the A segment. Analysis of its GPC chromatogram showed a unimodal peak, implying that there are no unconjugated segments [24,38,46,53,55].

Often, miktoarm polymer development is carried out using polymerization from heteromultifunctional cores. When there are equivalent initiating functional groups present in multifunctional or polymer segment-conjugated cores, it is generally assumed that the polymerization is initiated from every such functional group present. For example, in the development of a PEG-PLA_2_ miktoarm polymer from a PEG macroinitiator conjugated to a serinol core, L-Lactide ROP was initiated from both hydroxyls present in the core [34]. In such cases, GPC is used to verify A and B segment addition. The ^1^H NMR is then used to verify the overall polymer structure and calculate the degree of polymerization (DP) of A and B arms [32,34,40,43,44,54,62,65,67,72,74,116]. Such strategies have been applied to other miktoarm star architectures, including A_2_B_2_ and A_3_B_3_ [50,51].

Depending on the core structure, ^1^H NMR spectra can also provide more accurate confirmation of initiator usage, as with the disappearance of the corresponding protons [59,63]. For example, ethyl-β-d-glucopyranoside, a sugar (containing one secondary and three primary hydroxyls) was used to initiate the ROP of caprolactone, specifically at its secondary hydroxyl group, with the aid of the catalyst Novozyme 435. After the hydroxyl group was terminated with vinyl acetate, caprolactone ROP was initiated from the remaining hydroxyl groups, using a Sn(Oct)_2_ catalyst. PEG was then conjugated to free PCL-OH ends. In addition to standard GPC characterization, ROP from specific sugar hydroxyl groups was verified, due to their inequivalent ^1^H NMR peak shifts. While not a quantitative measure, FT-IR can also be used to verify the presence of functional groups derived from each arm composing a miktoarm polymer [52,58,73,78].

In one unique example, partially deprotonated PEG was used to initiate the ROP of glycidol to yield a short hyperbranched oligoglycidol with 10 hydroxyl groups for the ROP of caprolactone, which was followed by integration of the ^1^H NMR spectra. Based on the assumption that each hydroxyl group will be active, caprolactone ROP was subsequently carried out to yield the 10 PCL arms, and verified again using both NMR and GPC [37].

## 3. Amphiphilic Miktoarm Star Polymers: Self-Assembly

**Figure 4:**
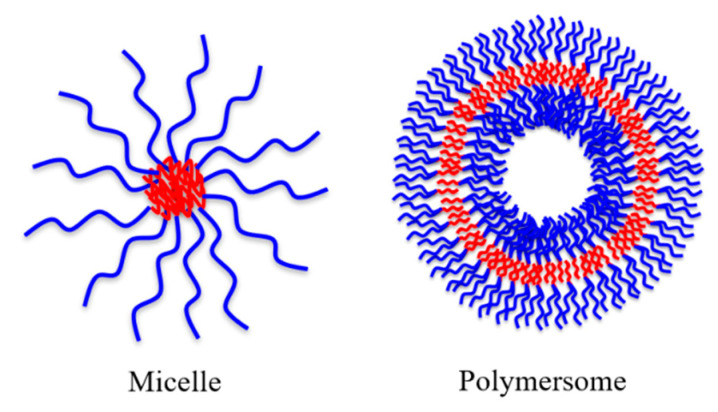
Aqueous self-assembly of AB_2_ miktoarm polymers into micelles and polymersomes, depending on polymer hydrophilic fraction (red = hydrophobic, blue = hydrophilic).

As discussed earlier, most pharmaceutical agents have inadequate bioavailability when administered directly [2,3], and thus, polymeric nanocarriers provide an important tool for drug delivery. Amphiphilic block copolymers and lipids have been extensively studied as potential platforms for loading and delivering drug cargo to targeted diseased sites within the body [7,117]. When introduced into aqueous media, the exposure to the newly polar environment forces amphiphilic polymers to undergo microphase separation, during which chains segregate into distinct polar and non-polar phases [118]. The overall assembly that results from the sequestering of chains, is generally guided by the total fraction of each polar/non-polar chain segment within the amphiphilic polymer, as well as its overall topology [119]. Although conditions vary, it is commonly accepted that for micelle formation, an amphiphilic polymer must have a hydrophilic fraction *f* > 0.45, and for polymersomes, *f* = 0.35 ± 0.1 (Figure 4) [120,121]. While micelles are characterized by their hydrophobic cores and hydrophilic coronae, polymersomes distinctly have a hydrophilic core, surrounded by a hydrophobic layer, enclosed within a hydrophilic corona. These domains make such nanostructures suitable for the physical encapsulation, prolonged retention, and delivery of poorly-water-soluble drugs, thus increasing their bioavailability and overall therapeutic efficiency. Some of the important parameters typical of nanocarriers used for drug delivery applications include their size, CAC/CMC, biocompatibility, drug loading, and drug release [8,25,122]. While much of the familiarity surrounding the effects of polymer self-assembly on drug delivery is derived from work on amphiphilic linear diblock copolymers, miktoarm star polymers with asymmetric branching polymer segments have increasingly been shown to possess superior micelle properties, while also being more tunable, due to their varied syntheses and number of unique constituent segments [21,122].

As discussed earlier, micelle diameters below 200 nm are necessary to improve their biological distribution and disease site accumulation, commonly through the EPR effect [12]. Self-assembled structures from miktoarm star polymers can, in general, have similar sizes to those from linear block copolymers [52], and scale similarly with increasing hydrophobic segment size relative to hydrophilic blocks [24,56,62]. The branched architecture of miktoarm stars offers potential for tuning, and this was explored in a study of three copolymers using biocompatible PEG and PCL arms, with different AB, BAB, and AB_2_ (A = PEG, B = PCL) diblock, triblock, and miktoarm topologies, but with similar hydrophilic/hydrophobic ratios [60]. XRD was used to study PEG and PCL crystallinities, and it was found that the relative crystallinity of PCL chains decreased in the sequence AB > BAB > AB_2_, as the arms became more sterically restricted. PEG crystallinity was found to be severely limited in the BAB triblock as a result of the adjacent PCL arms, and it was similar in AB and AB_2_ copolymers. This was further evidenced by the measurement of C=O vibrations using FTIR at 1726 and 1736 cm^−1^, for crystalline and amorphous PCL regions, respectively. Such variations in crystallinity accounted for the differences in micellar diameters, where AB, BAB, and AB_2_ copolymers had sizes of 43, 74, and 53 nm, respectively. On the other hand, one can design miktoarm star polymers to have a larger proportion of hydrophilic to hydrophobic arms, so that the relatively large volumes of the hydrophilic groups force a constricted micellar curvature, resulting in smaller diameters [123,124]. An AB_3_ (A = PMMA, B = PNIPAM) miktoarm star for prednisone acetate delivery was designed for this purpose, and it formed spherical micelles of roughly 50 nm, considerably lower than the 190 nm diameters of the equivalent linear diblock analogues [31,125]. Depending on the polarity of the majority of branches, miktoarm polymers can have different properties. Increased hydrophobic branching can diminish core crystallinity, due to imperfect packing, with an insignificant increase in size, and such an effect has been demonstrated to be beneficial for drug loading [126]. 

### 3.1. Micelle Characteristics: CMC and Stability

There are several methodologies that have been employed for studying aqueous self-assembly of amphiphilic polymers, including co-solvent evaporation, thin-film, dialysis, and oil/water emulsion methods [127]. In the co-solvent evaporation and dialysis methods, the amphiphilic polymer is solubilized in a miscible organic solvent, added to water slowly, and then the organic phase is removed through evaporation or dialysis, triggering self-assembly. In the oil/water emulsion method, the polymer is dissolved in a water-immiscible organic phase, followed by the addition of water, and evaporation of the organic phase. Regardless of the method, the lowest concentration at which the hydrophobic segments of amphiphiles will begin to sequester is known as the critical aggregation concentration (CAC), or the critical micelle concentration (CMC) for micelles. Amphiphilic polymers in solution exist as unimers below their CMC and partition to the air/solution interface. Continuously increasing polymer concentrations past the CMC leads to the formation of a separate phase composed of the self-assembled polymers, which is accompanied by a decrease of free energy of the system [128]. Due to the large dilution that polymeric micelles undergo upon administration into the body, having a very low CMC not only represents the general stability of micelles, but it is integral in maintaining their morphology, and in preserving their function [4].

The methods for determining a polymer’s CMC include tensiometry, conductometry, and fluorescence spectrometry, which measures the absorbance and emission spectra of encapsulated hydrophobic dyes. The onset of CMC is seen as a sudden shift in the rate of change of a measured variable as a function of polymer concentration [128]. The CMCs of miktoarm star polymer-based assemblies are often determined through fluorescence spectroscopy, using the hydrophobic fluorophore pyrene, or somewhat less commonly, Nile Red. With pyrene, for example, its partitioning from the aqueous phase (below the CMC) towards micelle interiors (above the CMC), is reflected by an increase in fluorescence intensity and a red-shift of its (0,0) vibronic band [129]. Consequently, the CMC can be found as a change in the intensity ratio of I_338_-I_339_/I_333_-I_336_ in the excitation spectra [51,130]. Importantly, the CMCs attained in miktoarm star assemblies (10^−7^–10^−9^ M) have generally been found to be lower than those of diblock copolymers (10^−4^–10^−7^ M), though it should be noted that most diblock systems have CMCs closer to 10^−7^ M [4,14,15,16,17,20,21,22,23,24,25,75]. Incorporation of additional arms in miktoarm star polymers allows for further stabilization of micelles, leading to lower CMCs. This normally involves tailoring miktoarm stars to have a larger proportion of hydrophobic to hydrophilic segments, so that self-assembly is more energetically favorable. For example, in a study comparing AB and AB_2_ (A = PEG, B = poly(glutamic acid (PGA)) polymers with conjugated DOX, the miktoarm star CMCs were lower at 5.4 mg/L compared to the diblock polymer’s 9.0 mg/L [52]. Similarly, AB_3_ miktoarm star polymers (A = PMMA, B = PNIPAM) were found to have considerably lower CMCs of 10 mg/L than their diblock equivalents (50 mg/L) [31,125].

With miktoarm polymers, as in the case of more conventional block copolymers, CMCs tend to decrease with increasing hydrophobic chain length. This is generally attributed to the favorable de-solvation and aggregation of hydrophobic segments in aqueous media [131]. It has been well documented for miktoarm star polymers, especially those containing PCL, in varied systems, including ABC (PEG-PCL-TPPBr) (triphenylphosphonium bromide) and PEG-PCL-PAA, as well as more complex A(AB)_3_, A_2_(AB)_2_, and A_3_(AB) (A = PEG, B = PCL) systems [42,56,57]. One interesting example was of an AB_2_ (A = PEG, B = P(MAA-co-MMA) miktoarm star, where poly(methacrylic acid) (PMAA) was a pH-responsive unit. Polymers containing higher hydrophobic PMMA to hydrophilic PMAA content ratios led to more compact and stable micelles and lower CMCs, with the trade-off that they were comparatively less pH-responsive [62]. While hydrophobicity tends to decrease CMCs, they generally increase with increasing hydrophilic block sizes [56,72].

Rather than strictly increasing hydrophobic block sizes, one study attempted to change the ratio of hydrophilic and hydrophobic segments through four different A_2_B, A_2_B_2_, AB_3_, and A_3_B (A = PCL, B = POEGMA) miktoarm star polymers [70]. It was found that increasing the number of PCL arms to POEGMA resulted in the largest increase in micelle stability, with a minimum CMC of 2.66 mg/L for the A_3_B miktoarm star. Interestingly, despite PCL/POEGMA ratios, the 4-armed miktoarm stars collectively had lower CMCs than the 3-armed A_2_B miktoarm star. This reciprocated an earlier study where more arms in star polymers increased micelle stability [132]. Similarly, the 3-armed star had the highest diameter of 73 nm, and the size decreased with the number of PCL arms in 4-armed miktoarm star polymers to 28 nm.

### 3.2. Micelle Drug Loading and Release

As mentioned earlier, most pharmaceutical agents have poor water-solubility and low bioavailability [2,3]. Loading drugs into self-assembled polymeric micelles can help resolve this issue by providing solvation, enabling prolonged gradual release, and through the EPR effect, facilitate passive targeting to disease sites [7,12,117]. Owing to their tailorable architecture, superior CMCs, improved drug loading, and sustained drug release, there has been much recent interest in miktoarm star polymer-based assemblies for drug delivery [21,25,26]. Drug incorporation into micelles has been shown to have varied effects on the size of miktoarm micelles, where it can lower, increase, or have a negligible effect upon drug encapsulation [24,42,47,48,49]. Though generally, a decrease in size has been attributed to good polymer-drug compatibility [47,49,57]. One of the beneficial effects of including multiple branching arms as in miktoarm stars, is in increasing drug loading content into their self-assemblies. For example, AB_3_ (A = PMMA, B = PNIPAM) self-assembled miktoarm polymers were used to load prednisone acetate with encapsulation efficiencies of 50%, compared to 11% with the corresponding linear diblock copolymers [31,125]. Improved drug loadings were also seen in AB_2_ type miktoarm stars with A = PNIPAM, B = poly(undecylenic acid) (PUA), and A = PEG, B = poly(trimethylene carbonate) (PTMC) [27,32,133].

Micellar drug retention has generally been associated with more sustained drug release and dampening burst release [32,48,55,70], which has the benefit of increasing drug bioavailability. This could be the result of strong interactions and more sites of association between the encapsulated drug and multiple hydrophobic chains [27,52,70,133]. In a study of A(AB)_3_, A_2_(AB)_2_, and A_3_(AB) (A = PEG, B = PCL) miktoarm stars, camptothecin loading percentages ranged from 3.6 to 10.8%, with higher loadings in assemblies of miktoarm stars with more PCL blocks [57]. A higher degree of branching in polymer-based nanocarriers is imperative in improving their therapeutic efficiency.

It has been argued that the branching architecture of miktoarm stars has favourable effects on drug loading and release properties, and increasing hydrophobic chain length can lead to an increase in drug encapsulation and prolonged drug release [32,48,62]. However, long hydrophobic segments may also prohibit micellar hydrophilic surface coverage [13,52,134]. This has been mainly a concern for diblock copolymers, and it can be lessened by making use of the branched architectures. For example, in a study of AB_2_ (A = poly(lactide-co-glycolide) (PLGA), B = PEG) miktoarm stars, increasing the length of PLGA arms was shown to result in total cumulative ibuprofen release to be between 10 and 60% [75]. In another study (using AB_2_ (A = PCL, B = PEG) miktoarm stars), nimodipine loading efficiencies, scaled between 23 and 70%, with variations in PCL length in PEG775_2_–PCL5800 and PEG775_2_–PCL19000. However, the drug release was found to be between 93 and 85% of their loaded cargo, respectively, showing that PCL size or drug loading efficiency had little effect on drug release [24]. Interestingly, increasing the hydrophilic chain length of a miktoarm star polymer can also promote drug loading, as was shown for a miktoarm star with two hydrophilic PNAM chains and hydrophobic poly(L-lactide) (PLLA) arm [72].

### 3.3. Non-Spherical Micelles

Due to a wide range of the hydrophilic fractions (*f* > 0.45) that conventionally permit amphiphilic polymers to form spherical micelles, and as a result of their simple morphology, micelles constitute the bulk of assemblies that have been explored for drug delivery. However, through the alteration of hydrophilic fractions of constituent amphiphilic polymers, it is possible to assemble nanocarriers with different morphologies. When the hydrophilic fraction is lower than 0.45, polymers tend to form polymersomes, inverted nanostructures, and cylindrical micelles, amongst other morphologies [120,121]. This rule is not universally followed, as for example, in a study with AB (A = PEG, B = PCL) type diblock copolymers, spherical micelles were obtained with PEG fractions of 0.5, 0.3, and 0.17. AB_2_ polymers with hydrophilic PEG fractions of 0.55, 0.32, and 0.20, instead formed fiber-like cylindrical micelles at *f* ≤ 0.32 [54]. Generally, the transition from spherical to cylindrical micelles occurs as the hydrophilic fraction of the constituent polymers decreases, as a result of a change in the curvature. The increase in the size of the hydrophobic segment decreases the interfacial curvature, resulting in fiber-like micelles. Unlike linear AB block copolymers, the miktoarm stars transitioned to form cylindrical assemblies at higher hydrophilic fractions as a result of the lateral crowding of PCL arms. Additionally, owing to their branching, miktoarm stars had generally lower CMCs except in the case of the 0.2 fraction polymers; and higher DOX loading efficiencies, reaching 73%, as opposed to 55% for diblock co-polymers. A comparison of DOX release between the cylindrical *f*_PEG_ = 0.32 micelles and spherical 0.30 micelles, showed 27 and 48% cumulative release, respectively. Another AB_2_ (A = PEG, B = PCL) miktoarm star containing a β-aminoacrylate junction, was found to form spherical micelles at *f*_PEG_ = 0.71, cylindrical micelles at *f*_PEG_ = 0.56–0.33, and platelet-like structures for *f*_PEG_ = 0.23. Interestingly, loading the *f*_PEG_ = 0.56 polymers with chlorin-e6 (Ce6) and DOX resulted in a spherical morphology [74].

Rather than assembling a structure defined by fractional hydrophilicity, (AB)_2_C_2_ (A = PS, B = poly(vinylidene fluoride) (PVDF), C = PEG) miktoarm stars were observed to self-assemble into 99 nm micelles in aqueous solution with distinct wrinkled cores [69]. PVDF in the micellar inner core blocks had both lipo-philicity and -phobicity. Cryo-TEM evaluation revealed that micelles had a “frustrated” wrinkled core, due to the resultant immiscibility of PVDF and PS. As a proof of concept for drug delivery, these micelles were loaded with Nile Red, and due to imperfect packing of PS and PVDF, the cargo could be encapsulated in empty pockets within the core, potentially allowing for high loading of drugs.

### 3.4. Polymersomes

**Figure 5:**
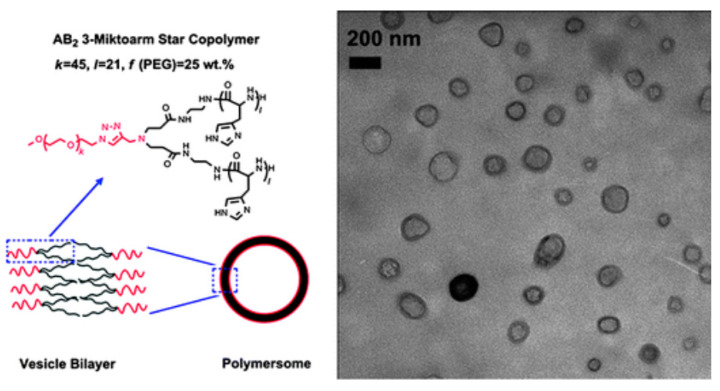
Cartoon schematic (**left**) and comparative TEM micrograph (**right**) of a polymersome assembled from a PEG-PHis_2_ miktoarm star polymer with emphasis on its hydrophobic bilayer. Reprinted with permission from reference [43]. Copyright 2012 Royal Society of Chemistry.

While micellar systems have been the main focus of miktoarm star-based formulations for drug delivery, polymersomes remain an interesting and valuable option. This is due to the fact that they can load both hydrophilic and hydrophobic drugs as a result of their unique morphologies. Part of the reason why polymersomes have been more seldom explored in drug delivery is that amphiphilic polymers, including miktoarm polymers, are synthesized with hydrophilic/hydrophobic ratios that favour micelle formation upon self-assembly. As stated earlier, hydrophilic fractions of 0.35 ± 0.1 generally favor the formation of polymersomes [120,121]. Such approximations are difficult to apply universally, especially when considering the variably branched architectures of miktoarm polymers. A comparison of AB and AB_2_ (A = PEG, B = PLLA) polymers with varying hydrophilic PEG volume fractions revealed that self-assembly of miktoarm star polymers into polymersomes, is much more tolerant of more varied volume fractions. Polymersome formation was observed for PEG volume fractions of *f* = 0.2–0.7, much broader than the range of 0.2–0.4 for the linear diblock counterparts [34]. In another example, an AB_3_ (A = PAzo, B = PEG) miktoarm star polymer with an azobenzene-containing 4-isobutyloxyazobenzene side chain on the polymethacrylate arm, had a 78/22 hydrophobic/hydrophilic ratio, slightly outside the typical range for polymersome formation, yet it was still well suited for polymersome assembly. Although their spherical nature can be easily established using electron microscopy or a combination of DLS and SLS, it is often necessary to specifically distinguish them from spherical micelles. To this end, TEM and cryo-TEM micrographs can determine whether particles are polymersomes, due to the visual presence of thin outer membranes (Figure 5) [43,53].

An AB_2_ (A = PEG, B = PCL) miktoarm polymer-based polymersome was used for the dual loading of both hydrophobic curcumin and hydrophilic methotrexate HCl, with encapsulation efficiencies of 14.13 and 10.93%, respectively. The nanocarriers showed a large burst release of methotrexate HCl unless co-loaded with curcumin. Curcumin was found to have an additional effect in combating multidrug resistance, as a small loading of it had a significant effect on enhancing methotrexate cytotoxicity [71]. Thus far, there has not been enough research to determine whether miktoarm architectures promote drug encapsulation, specifically in polymersomes. However, in one study, linear AB and miktoarm star AB_2_ and A(BA)_2_ A = PEG, B = PCL) polymer-based assemblies were used to load rhodamine B isothiocyanate-Dextran (RhDex), with encapsulation efficiencies of roughly 45 and 40% for their AB and AB_2_ polymers based systems, respectively. It showed good consistency between the two architectures, but with higher loading in the diblock. On the other hand, complete drug release occurred at 9 and 14 days for the AB and AB_2_ polymers, respectively, suggesting enhanced drug retention conferred by branching [40].

Trends relating to the physical properties of polymersomes do show some differences to those in micelles. For example, an increase in hydrophobic block size had the effect of hindering drug release, even in the case of those loaded in the hydrophilic core [34]. In comparing polymersomes of AB and AB_2_ (A = PEG, B = PLLA) type architectures, CACs were found to be consistently lower for polymersomes assembled from miktoarm polymers, and were also found to decrease with hydrophobic block size [34]. Though conversely, in a study using AB and AB_2_ (A = PEG, B = PCL) polymers, it was found that there was a small free energy (Δ*G*°) penalty associated with sequestering more PCL arms into the hydrophobic polymersome bilayer, and consequently, AB_2_ stars had slightly higher CACs than AB diblock copolymers [40].

An interesting property of polymersomes is that they are capable of mimicking the architecture of cellular phospholipid bilayers. Assembly of branched AB_2_ miktoarm polymers as structural components in polymersomes can better replicate these cellular phospholipid bilayers [43]. A study compared AB_2_ (A = PEG, B = polyhistidine (PHis)) miktoarm stars to liposomes and incorporated cholesterol into the assembled polymersomes to enhance stability [44]. A combination of DLS and SLS studies showed that loading these polymersomes with 1 and 5% wt. cholesterol, increased their size from 72 to 92 to 129 nm, while maintaining their polydispersity index (PDI) and morphology. Additionally, polymersome half-life increased considerably to 15 h from 1 h in blood plasma concentrations of bovine serum albumin (BSA). The incorporation of cholesterol also increased the rigidity of the polymersome interface, slightly delaying the release of 5(6)-carboxyfluorescein, and these nanoparticles showed better cellular uptake.

## 4. Drug Delivery

Aqueous self-assembly of amphiphilic asymmetric branched architectures has intrigued the scientific community, due to its potential in tuning core-corona architectures in the resulting aggregates. It has been demonstrated that, compared to linear diblock copolymers, which have been extensively employed in drug delivery, the branching architecture of miktoarm star polymers affords enhanced micellar stabilities, lower CMCs, and could encapsulate hydrophobic drugs with much higher efficiencies and loading capacities [21,22,23,25,54,67]. Asymmetric arm build-up of miktoarm stars has promoted the development of several unique systems that take advantage of having multiple polymer arms, each with specific effects. For example, using high yield reactions and a combination of ring-opening and stitching methodologies, a variety of AB_2_ (A = PCL and B = PEG) and ABC (A = PCL, B = PEG, and C = PS) type miktoarm polymers have been prepared through the design of cores on which orthogonal reaction sequences could be easily carried out [24,39]. Using imaging studies, intracellular localization into lipid droplets has also been demonstrated, and the niacin conjugated nanostructures suppressed bacterial endotoxin stimulated nitric oxide production in microglia [41].

**Figure 6:**
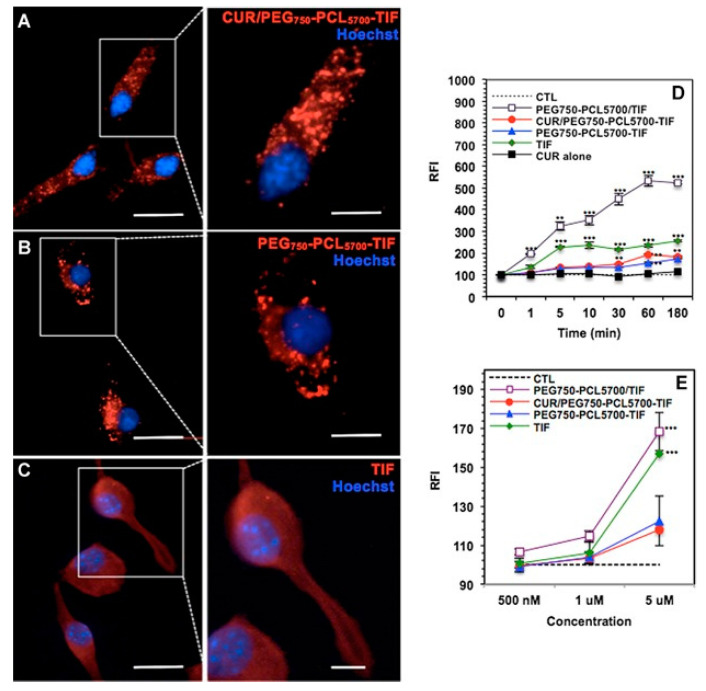
Fluorescent micrographs of macrophages treated with (**A**) curcumin-loaded PEG-PCL-TIF micelles, (**B**) unloaded PEG-PCL-TIF micelles, and (**C**) TIF control. Relative fluorescence intensities of macrophages treated with control media, TIF-loaded PEG-PCL micelles, curcumin-loaded PEG-PCL-TIF micelles, unloaded PEG-PCL-TIF micelles, free TIF, and free curcumin as a function of (**D**) treatment time and (**E**) dose concentration. Scale bar = 20 μm, inset scale bar = 10 μm. Statistically significant differences are represented by ** (*p* < 0.01), and *** (*p* < 0.001). Reprinted with permission from reference [49]. Copyright 2014 John Wiley and Sons.

In another study, tetraiodofluorescein (TIF), a fluorescent dye, containing ABC (A = PEG, B = PCL, C = TIF) miktoarm star was synthesized, by sequential click coupling of mPEG and propargylated TIF to a central core, and ROP of caprolactone on the third junction [49]. The miktoarm star polymers formed micelles in an aqueous medium with a diameter of about 115 nm and a CMC of 0.43 mg/L. These micelles showed a cumulative release of 66% over seven days. When macrophages were treated with them and imaged using fluorescence microscopy after labeling them with Hoechst 33342, the nanocarriers were clearly visible upon uptake into the cells (Figure 6). The nano-delivery system was found to decrease LPS-induced nitric oxide release in stressed macrophages, demonstrating their anti-inflammatory properties.

A series of mitochondria-targeting ABC (A = PEG, B = PCL, C = triphenylphosphonium bromide (TPPBr)) miktoarm star polymers were developed for coenzyme Q10 (CoQ10) delivery [42]. The star polymers with molar masses ranging from 6000 to 12,000, formed micelles with a size range of 26.7–43 nm, and encapsulation efficiencies of 83–85%. Fluorescein isothiocyanate (FITC) labeled miktoarm stars-based micelles with the TPPBr functionalities at the corona surface showed significant localization in mitochondria in neurons and glia cells, compared to polymers without any mitochondria targeting moiety. Furthermore, MTT assays and confocal micrographs showed that miktoarm stars carrying CoQ10 improved mitochondrial metabolic activity within 24 h and reduced mitochondrial damage from reactive oxygen species (ROS) in primary hippocampal cultures.

Miktoarm polymers could also be designed to lead to more stable self-assembled micelles by including stereochemically opposing arms as hydrophobic segments [35]. In this regard, AB_2_ and ABC-type miktoarm star polymers were synthesized based on cyclic carbonate-functionalized PEG, on which ring-opening of the carbonate was carried out with an amine-functionalized silyl protecting groups. The order and extent of ROP could be controlled to yield miktoarm stars with a combination of poly(D-lactic acid) (PDLA) and PLLA- based hydrophobic segments. The linear diblock analogs of these, which were composed of PEG and either PLLA or PDLA, had CMCs of 24.0 and 25.1 mg/L. The addition of another hydrophobic segment (PLLA or PDLA) in the branched miktoarm polymers, reduced these CMCs to 19.1 and 20.0 mg/L. The branched miktoarm architectures with both PLLA and PDLA as hydrophobic arms had opposing stereochemistry, allowing stabilization through complexation within the core. This had the effect of further reducing CMCs to 15.1 mg/L. The introduction of these stereochemically opposing arms also led to the reduction in size from 221.6 nm (for PEG-PDLA-PDLA) to 160.0 nm (for PEG-PLLA-PDLA). With paclitaxel as a model drug, the miktoarm micelles showed a cumulative release of 55% over 10 days. Stereocomplexation was also demonstrated to promote a much more sustained release of about 35% over this time period.

### 4.1. pH-Responsive Drug Delivery

The design of drug delivery nanocarriers often involves careful tailoring of polymer constituents for enhanced micellar stability, and the introduction of endogenous stimuli-responsive functional groups. For example, there is a considerable variation of pH in the blood, tissue, stomach (1.5–3.5), the small intestine (5.5–6.8), the colon (6.4–7.0), as well as in the intracellular endosomal (5.5–6.0) and lysosomal (4.5–5.0) environments. One could use pH stimulus in drug delivery, and the constituent polymers must be resistant to degradation in these environments, and must have a pK_a_ adapted to targeted delivery [135]. Polymers used for pH-responsive drug delivery, such as PDEAEMA or poly(methacrylic acid) (PMAA), are often weakly basic. It should also be noted that, while not inherent to nano-delivery systems, acidic conditions can also increase drug release from miktoarm polymers, due to drug protonation, resulting in increased aqueous solubility [72].

An A_3_(BC)_3_ (A = PCL, B = PDEAEMA, C = poly(poly(ethylene glycol) methyl ether methacrylate) (PPEGMA)) miktoarm polymer was synthesized by combining ROP of caprolactone, and sequential ARGET-ATRP of DEAEMA and PEGMA, using a multifunctional dipentaerythritol core [51]. PCL was the biocompatible hydrophobic block that would form the core of self-assembled micelles, PDEAEMA was the pH-responsive entity, which at a pH lower than 6.9 became protonated, leading to hydrophilic/hydrophobic switch. The PPEGMA block provided a hydrophilic shell, which imparted biological stability. At pH > 8, the PDEAEMA chains were found to be completely deprotonated, and as a result, collapsed to form the hydrophobic core with PCL. A decrease in pH to 4 led to a gradual increase in micelle size, due to both PDEAEMA chain expansion upon becoming soluble, and electrostatic repulsion between the now positively charged chains. At pH of 7.4, 6.5, and 5.0, TEM micrographs showed that micelles had diameters of 100–180, 250–350, and >500 nm, respectively. DOX-loaded A_3_(BC)_3_ star-based micelles with an encapsulation efficiency of 42–71%, showed steady drug release curves, culminating in 27–40% over 96 h. Decreasing the pH of the release medium to 6.5 and 5.0 increased the cumulative release to 44–59% and 85–100%, respectively. Generally, micelles with larger PDEAEMA fractions showed a greater response to pH, as one would expect. A_2_(BC)_2_ (A = PCL, B = PDEAEMA, C = PPEGMA) stars were also synthesized by the same group, and these polymers self-assembled to slightly smaller (63 nm) micelles [50]. Drug release at pH = 7.4, 6.5, and 5.0 showed cumulative DOX release of 82%, 50%, and 36%, respectively. While the cytotoxicity of unloaded micelles was negligible, DOX-loaded micelles exhibited similar anti-tumor efficiencies to free DOX in HepG2 cancer cells.

**Figure 7:**
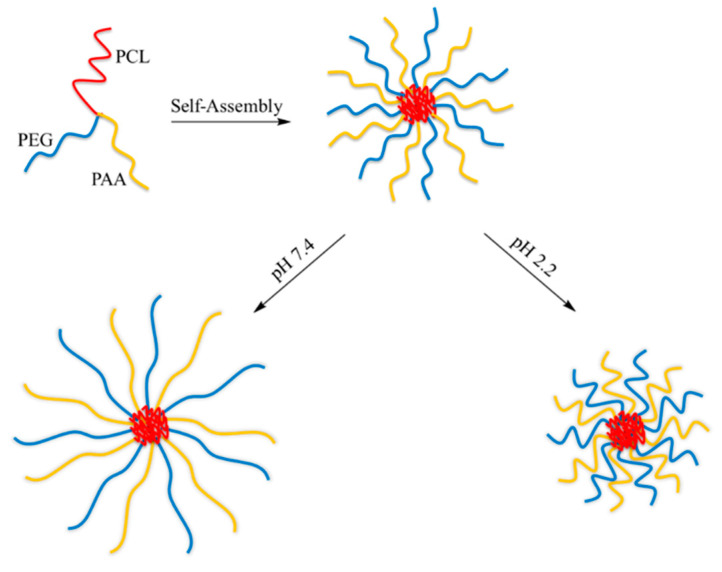
Representation of PEG-PAA-PCL miktoarm polymer self-assembly and pH-responsive morphological changes.

A series of ABC (A = PEG, B = PCL, C = PAA) star polymers that would assemble into micelles with hydrophobic cores and mixed PEG/PAA hydrophilic coronae with pH sensitivity imparted by PAA (Figure 7), have also been synthesized [56]. At low pH, the COOH group on the backbone of PAA arms became increasingly protonated and resulted in hydrogen-bonding complexation with PEG arms in micellar coronae, and a decrease in micelle size. In the pH range from 6 to 10, PAA arms became progressively ionized, and formed larger swollen micelles, from the loss of PEG/PAA corona complexation and repulsive forces between adjacent PAA arms. The average particle size increased from 51 to 154 nm at pH 2.2 to 10. The ABC miktoarm polymers had 58–73% encapsulation of naproxen, a model hydrophobic drug, and its cumulative release from the ABC miktoarm star micelles was 65–89% over 24 h, at pH 7.4. Adjusting the pH to 2.2, and therefore, compressing the micelles, resulted in greater drug retention, with cumulative naproxen release coming to roughly 35–50%. This suggests that naproxen would be retained under gastric conditions and released in the intestine, making the micelles suitable for oral administration.

A miktoarm star with 8 arms, A_2_B_6_ (A = PCL, B = PMMA-*co*-PMAA was prepared using D-(−)-salicin (a *β*-glucoside) as a heterofunctional initiator [63]. It was functionalized with six bromoester groups and two hydroxyl groups, and the miktoarm star was constructed using ROP of caprolactone and ATRP of tert-butyl methacrylate (tBMA)/MMA, initiated by the hydroxyl and bromoester groups, respectively. The t-butyl groups were then removed through acidolysis to give PCL_2_(PMMA-*co*-PMAA)_6_ stars. Upon self-assembly, the CMC was reported to be as low as 15 mg/L, yet in some samples, an additional higher point was found at 411 mg/L. TEM micrographs revealed the presence of dandelion-shaped superstructures with coronae surrounding dark spheres above the CMC. Two anticancer drugs, DOX and camptothecin (CPT), were loaded into the micellar structures with encapsulation efficiencies of up to 60% and 6%, respectively, and the drug-loaded micelles were found to be larger than 200 nm in diameter. Due to the pH sensitivity of PMAA, the release rates of both drugs were slower at pH 7.4 than pH 5.0, at which point the chains became ionized and caused micelle swelling.

Another example of a miktoarm star drug delivery system using PMAA as a pH-responsive unit had the composition of AB_2_ (A = PEG, B = P(MAA-*co*-MMA) [62]. The polymer was synthesized by conjugating a bromoisobutyrate ATRP initiator to a dihydroxy benzoic acid core, followed by mPEG-OH coupling, and ATRP of MMA and tBMA. Acidolysis with TFA afforded the PEG-P(MAA-*co*-MMA)_2_ miktoarm star polymer. The MAA content of the hydrophobic block was carefully controlled during copolymerization to tune the pH-sensitivity of miktoarm stars and two PEG-P(MMA_9_-*co*-MAA_35_)_2_ and PEG-P(MMA_24_-*co*-MAA_25_)_2_ miktoarm stars were synthesized. Higher MMA content led to higher (82.3%) encapsulation of methotrexate (MTX), an antineoplastic agent with significant chemotherapeutic activity, and the micelles exhibited a greater pH-induced MTX release (98% release after 48 h at pH 1.2), compared to 33% at pH 7.4.

**Figure 8:**
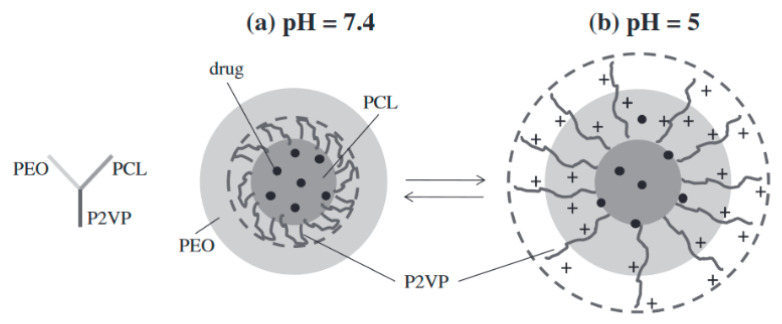
Schematic representation of the effect of (**a**) physiological pH and (**b**) acidic pH on the morphology of self-assembled PEG-PCL-P2VP micelles. Reprinted with permission from reference [29]. Copyright 2012 Elsevier.

Taking advantage of poly(2-vinylpyridine) (P2VP), containing basic units with a pK_a_ of roughly 5.9, a variety of pH-sensitive ABC-type (A = PEG, B = PCL, C = P2VP) miktoarm stars were synthesized [28,29,30]. Due to the presence of P2VP, these polymers formed micelles with positive (+12.5 mV) zeta potentials upon aqueous self-assembly, thus indicating the presence of protonated P2VP arms in the coronae alongside PEG. Deprotonation through NaOH addition resulted in a micelle diameter change from 54 to 37 nm, due to the collapse of P2VP branches into micelle cores. This was attributed to the unique architecture of the star polymers, as well as the loss of electrostatically repelling chains in the corona (Figure 8). A titration indicated that the pK_a_ was 5.0, corresponding closely to that for P2VP, and was neutral at physiological pH [28]. In a follow-up study, P2VP blocks were biotinylated so that, upon administration, the labelled ABC micelles would contain biotin in the core [29]. Exposure to low pH in a tumor environment would protonate P2VP chains and shift conjugated biotin to the micellar corona, where it would aid in tumor targeting. Interestingly, the passive uptake effect of cationic micelles, due to electrostatic interactions with negatively charged cell surfaces is so effective that the inclusion of biotin was unnecessary for astrocyte and 9L cell internalization. Loading of micelles with Nile Red as a model hydrophobe verified their efficacy in drug delivery. While 24% sustained release was seen after 6 h at pH 7, an acidic pH 5, mimicking the endosomal environment, caused burst release with a cumulative release of 64% after 6 h.

Rather than using intrinsically pH-responsive polymers, linear and miktoarm star polymers of AB and AB_2_ compositions, respectively (A = PEG, B = PGA), were prepared with DOX conjugated via an acid-labile hydrazone linker [52]. To achieve this, ROP of benzyl-L-glutamate was carried out on mPEG with either one or two primary amino groups. This was followed by the ester-amide exchange aminolysis of the benzyl protecting groups on the PGA blocks, and then the linker was conjugated to neutralized DOX. The micelles were also loaded with DOX, and it was observed that miktoarm star polymers had nearly twice the drug loading, as compared to their linear analogues. This may be related to the increased number of sites in the PGA blocks for the association. pH-dependent DOX release was seen in both AB and AB_2_ polymer micelles as a result of acid-catalyzed hydrazone linker cleavage. While all micelles showed a similar 45% cumulative release within 72 h at pH 7.4, AB_2_ micelles showed 15% faster release compared to AB micelles at pH 5.0, and 25% faster release than micelles at physiological pH. These DOX release rates also contributed to a larger tumor-suppressive effect when micelles were incubated with HeLa cells.

Another use of the pH-responsive hydrazone functional group was in linking the constituents of an AB_2_ (A = oleic acid (OA), B = PEG) miktoarm star for antibacterial drug delivery [77]. The hydrazone linker itself was formed from the combination of a hydrazide functionalized G1 oleodendrimer with PEG-CHO, and its efficient hydrolysis was initially confirmed via incubation of the AB_2_ star in phosphate-buffered saline at pH 6.0. It resulted in fragmentation, observed using ESI, which showed peaks related to the cleaved oleic acid segment. The miktoarm star polymers self-assembled into 130 nm micelles with 6 mg/L CMC, and were used to load vancomycin, an antibiotic, with an encapsulation efficiency of 39.6%, showing good compatibility with the micellar core. Drug release experiments showed the benefit of the hydrazone linker, as the cumulative release of drug-loaded micelles reached 100% at pH 6.0 in 48 h, due to hydrazone linker cleavage (and subsequent micellar disassembly), as opposed to 86% release at pH 7.4. It allowed for antibacterial activity for 52 h when tested against *S. aureus*, while unloaded vancomycin was only effective for 18 h. The nanocarriers were also shown to be more effective in depleting bacterial growth in a skin infection model.

AB_2_ (A = PEG, B = PHis) type pH-sensitive miktoarm star polymers for self-assembly into polymersomes were prepared [43]. Using a 150 mM NaCl solution, it was found that the miktoarm star effectively buffers in a pH range of 5–7, with an effective pK_a_ of 6, which is in the endolysosomal pH range. Shifting pH from physiological pH at 7.4 down to 6.8, 6.0, and 5.0 was also associated with morphological transitions to cylindrical micelles, spherical micelles, and finally, soluble unimers. The polymersomes showed encapsulation of 5(6)-carboxyfluorescein, a fluorescent hydrophilic dye, similar to that for liposomes, and a drug release profile indicating about 70% release in 72 h. Decreasing the pH below 6.8, and thus triggering structural changes, resulted in a complete burst release within a few hours, and it showed good sensitivity to the endosomal pH range.

### 4.2. Temperature-Responsive Drug Delivery

Higher temperatures at disease sites provide another venue to target and control the release of encapsulated drugs from nanoparticles. In addition, heat can be applied from external sources in order to trigger the nanoformulation response. Polymers used in temperature-responsive systems typically feature a lower critical solution temperature (LCST) near the physiological temperature, above which a polymer becomes immiscible. The most common temperature-responsive polymer used with drug delivery systems, and the only one used in miktoarm star-based drug delivery systems is poly(N-isopropylacrylamide) (PNIPAM), due to its availability, biological compatibility, and well-studied LCST in the range of 30–35 °C [136].

In a very early example, an AB_2_ (A = PNIPAM, B = poly(undecylenic acid) (PUA)) miktoarm star was synthesized that had an LCST of 31 °C, very close to accepted values for PNIPAM [27]. Upon loading prednisone acetate, a common anti-inflammatory drug, it was reported that at temperatures below LCST, even after 150 h, more than 80% of the drug remained intact. However, significantly faster release rates were seen above the LCST of PNIPAM. In a later study, similar AB_3_ miktoarm star polymers (A = PMMA, B = PNIPAM) were synthesized [31], and below the LCST of PNIPAM, these formed spherical micelles in a size range of 50 nm, and with a CMC of 10 mg/mL. Prednisone acetate was stabilized in the PMMA cores of the micelles, and there was a total cumulative release of 55% at room temperature, compared to a 90% release above LCST. In such cases, the conversion of PNIPAM to a hydrophobic polymer above its LCST results in its collapse from the micellar corona into the core. As a result of the morphological transformation, an increase in the cumulative release of prednisone takes place [27,31].

**Figure 9:**
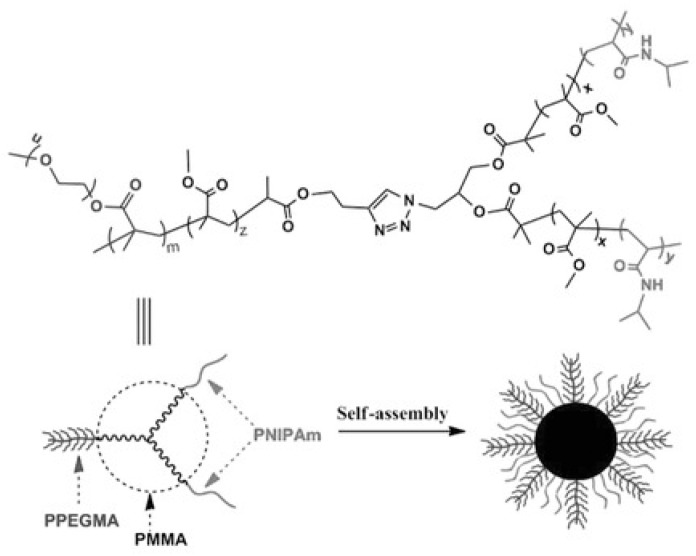
Schematic illustration of the micellar self-assembly of (BA)(AC)_2_ miktoarm star polymers. Reprinted with permission from reference [65]. Copyright 2016 John Wiley and Sons.

While the previous examples deal with the corona/core switch of PNIPAM, one could also stabilize its position in micellar coronae via crosslinking. Crosslinking the micellar shells of assembled AB_2_ (A = PNIPAM, B = poly(L-lysine) (PLL)) miktoarm star polymers would not lead to micellar collapse following the temperature increase above the LCST of PNIPAM, and the effect on drug delivery would be more subtle [38]. In such a system, shell crosslinking of drug-loaded micelles was carried out using glutaraldehyde, and the permeability of the shell could be controlled by the extent of its reaction. The effect of restricted shell permeability was seen through prednisone acetate release, where the cumulative release from 50 and 100% crosslinked micelles was 17.1 and 22.8% after 170 h. Increasing the temperature to 38 °C, above the LCST of PNIPAM, led to cumulative releases of 44.7 and 51.2% over the same time period, showing an accelerated yet still very sustained release.

(BA)(AC)_2_ (A = PMMA, B = PPEGMA, C = PNIPAM) miktoarm polymers were synthesized through a combination of CuAAC coupling with ATRP, on a 1-azido-2,3-propanediol core (Figure 9) [65]. These miktoarm stars contained only hydrophobic blocks emanating from the core junction, with hydrophilic chains being linked to hydrophobic PMMA chain-ends. While it was speculated that this structure may result in especially low CMCs, it was measured at 2 mg/L, in line with most other miktoarm star polymers. An interesting aspect of this miktoarm star architecture stemmed from the inclusion of PNIPAM arms, which, upon micellar self-assembly at room temperature, were found to be in the coronae of micelles alongside PPEGMA, but collapsed into the PMMA cores at physiological temperatures. When the temperature was further increased above the LCST at 42 °C, it resulted in micellar aggregation. Celecoxib, a hydrophobic drug, was loaded into the micelles with 8.8% encapsulation efficiency, and in 48 h, 73% of celecoxib was released from micelles at 25 °C. However, upon increasing the temperature to 37 °C, the release rate was found to be faster (89% drug release over 48 h). This was explained using the general effect of temperature on release kinetics, as well as dissociation of the drug from PMMA, as PNIPAM began to permeate the core.

### 4.3. Redox-Responsive Drug Delivery

Oxidative stress is characteristic of many pathologies, such as neurodegenerative disorders, cancer, and diabetes, which results in heightened concentrations of reactive oxygen species (ROS), including hydrogen peroxide (H_2_O_2_), superoxide (O_2_^−^), hydroxy radicals (·OH), and singlet oxygen (^1^O_2_) [137]. While normally present as regulators in redox-dependent signal transduction [138], elevated ROS concentrations at diseased sites are a sign of insufficient activity from the endogenous antioxidant defense mechanisms. One aspect of this system is the antioxidant glutathione (GSH), which is oxidized to glutathione disulfide (GSSG) upon exposure to ROS [139]. In cancer, tumor cells are known to contain elevated GSH levels that are thought to aid in tumor cell proliferation [139]. Additionally, evidence suggests that GSH protects cancer cells against chemotherapeutic drugs [140]. While a variety of cleavable polymer linkers have been applied to ROS-responsive drug delivery, including those based on thioketals, diselenides, phenylboronic acids, and esters, and vinyldithioethers [137], interestingly, only the β-aminoacrylate was used as a specifically ^1^O_2_-labile linker in miktoarm polymers [74]. Much effort has been devoted to developing miktoarm stars responsive to GSH that is present at elevated concentrations intracellularly at disease sites. In preparing GSH-responsive systems, polymers are typically conjugated using disulfide linkers that can be cleaved through thiol-disulfide exchange. GSH can also participate in thiol-thioester exchange, facilitating polymer cleavage, and it is reactive to acrylates via Michael additions, which can be used to prepare GSH conjugates [141,142].

An example of a miktoarm star containing a GSH-responsive linker was prepared using an AB_2_ (A = PEG, B = PMMA) build-up, based on a dihydroxy-benzoic acid core with cystamine, a disulfide linker, connecting the core and the hydrophilic PEG arm [58]. The dihydroxy functionalities on the core were coupled to bromoisobutyrate ATRP initiators and used to polymerize PMMA. Aqueous self-assembly of these polymers led to micelles with a diameter of 130 nm and CMC of 0.91 mg/L. As a substitute for GSH, which would be present biologically, 10 mM of dithiothreitol (DTT) was used (mimicking intracellular GSH concentrations) to reduce the disulfide linker present in the micelles. This treatment resulted in a micelle size shift to 300 nm after 2 h, and >1000 nm after 24h, as a result of the aggregation of unlinked polymer chains, while no significant size change was seen in untreated samples. MTX was encapsulated with 64% efficiency, and the nanocarriers showed a 22% release in 48 h with no clear burst release. Meanwhile, DTT treatment, causing complete micellar dissociation, resulted in a much more significant (95%) release in 48 h.

**Figure 10:**
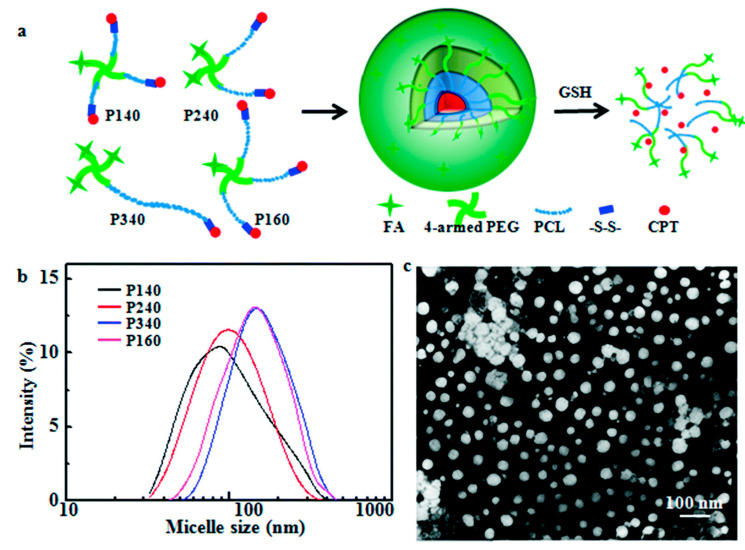
(**a**) Scheme of A(AB)_3_ (P140, P160), A_2_(AB)_2_ (P240), and A_3_(AB) (P340) assembly into micelles, their response to GSH, (**b**) their size distributions from DLS, and (**c**) a representative TEM micrograph of A_2_(AB)_2_ (P240) micelles. Reprinted with permission from reference [57]. Copyright 2015 Royal Society of Chemistry.

A series of miktoarm polymers based on a core with four branching PEG arms have been prepared, by selectively carrying out the ROP of caprolactone on individual PEG ends to yield A(AB)_3_, A_2_(AB)_2_, and A_3_(AB) (A = PEG, B = PCL) stars (Figure 10) [57]. PEG blocks were end-conjugated to folic acid (FA) while PEG-PCL ones were conjugated to camptothecin (CPT) via dithiodipropionic acid, a GSH-cleavable disulfide bearing linker. Incubation with GSH not only led to the cleavage of CPT conjugated directly to miktoarm stars, but also to a several-hundred nanometer increase in micellar diameter—which was associated with core destabilization from the linker cleavage. Faster CPT release was seen in micelles that had the highest CPT content (more points of conjugation), with 76%, 69%, and 54% release coming from A(AB)_3_, A_2_(AB)_2_, and A_3_(AB) micelles, respectively. Cellular uptake of CPT was investigated with SKOV-3 cells that overexpressed FA receptors. Micelles with the highest FA surface density (those with free PEG arms conjugated to FA) were expected to enable higher cellular uptake. The A_2_(AB)_2_ micelles promoted the CPT uptake the most at 25%, and 11 and 15–20% in A(AB)_3_ and A_3_(AB) micelles, respectively, suggesting a compromise between FA surface density and CPT-loading. Considering CPT loading, GSH-sensitivity, and cellular uptake, PEG_2_-(PEG-PCL)_2_ micelles were the most optimal as anti-tumor agents.

AB_2_ (A = PEG, B = PLL) type miktoarm stars have been prepared using N-carboxyanhydride (NCA) ROP on a PEG-based macroinitiator, with or without bioreducible disulfide linkages conjugating the polymeric arms [73]. The disulfide linker-containing system employed cystamine, which was conjugated directly to the PEG macroinitiator, and its NH_2_ end was employed for ROP. PLL is cationic at physiological pH, and it can electrostatically bind with negatively charged biomolecules, such as plasmid DNA (pDNA). It was demonstrated that a 5:1 wt. ratio of miktoarm polymer to pDNA was sufficient for complete binding. Miktoarm star/pDNA polyplexes were prepared using this ratio, and these formed spherical particles of 57.67 and 142.62 nm diameters, with or without reducible disulfide linkers, respectively. These sizes were constant when incubated with fetal bovine serum for 24 h, indicating good nanoparticle stability and applicability for intravenous administration. This was in contrast to complexes of pDNA with just PLL arms, which grew to about 450 nm after 24 h. The significance of the effect of the linkers or the reasons for the disparate sizes is not fully understood. Unlike the non-bioreducible polyplexes, pDNA bound to miktoarm stars was released in response to DTT, a reducing agent substitute for biological GSH. The *in vitro* transfection efficiency of miktoarm polyplexes containing disulfide linkers into HeLa cells, was observed to be much higher than that of the non-reducible polyplexes, due to the intracellular reduction of these linkers.

**Scheme 6:**
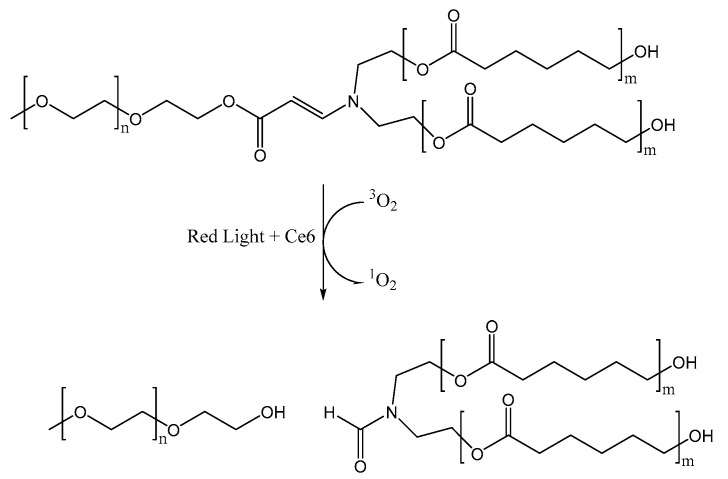
Structure and ^1^O_2_-response of PEG-PCL_2_ miktoarm star polymers with β-aminoacrylate junctions to 660 nm light in the presence of Ce6.

A ^1^O_2_-responsive miktoarm star of AB_2_ (A = PEG, B = PCL) composition has been prepared, using the ROP of caprolactone from a PEG macroinitiator, containing a β-aminoacrylate junction [74]. The latter was found to be 100% in the E-configuration, and its ^1^O_2_ induced cleavage was confirmed under red light laser irradiation, after exposure to the ^1^O_2_-generating photosensitizer, chlorin e6 (Ce6) (Scheme 6). Ce6 and DOX were loaded into the micelles with 78% and 29% efficiencies, respectively. Irradiation of these Ce6/DOX co-loaded samples led to the disruption of micelles into irregular aggregates, which triggered 68% DOX release after 24 h, as opposed to only 26% in the dark. Accordingly, laser irradiation of co-loaded AB_2_ micelles resulted in large DOX uptake into the cytoplasmic regions of MDA-MB-231 cells, more so than even free DOX, while micelles kept in the dark maintained good cargo retention.

### 4.4. Light- and Dual-Responsive Drug Delivery

Light is an advantageous non-invasive exogenous stimulus that can be directed to a target site, and it provides an intriguing platform for drug delivery. However, since UV light is often used to trigger such systems, safety risks, as well as low tissue penetration, are matters of concern. The two main categories of light-responsive polymers that have been explored in drug delivery are those that undergo photochemical cleavage or photoisomerization [143]. For such UV-light induced cleavage, moieties, such as pyrenylmethyl esters, coumarinyl esters, and *o*-nitrobenzyls, have been incorporated into polymeric systems [143,144].

**Figure 11:**
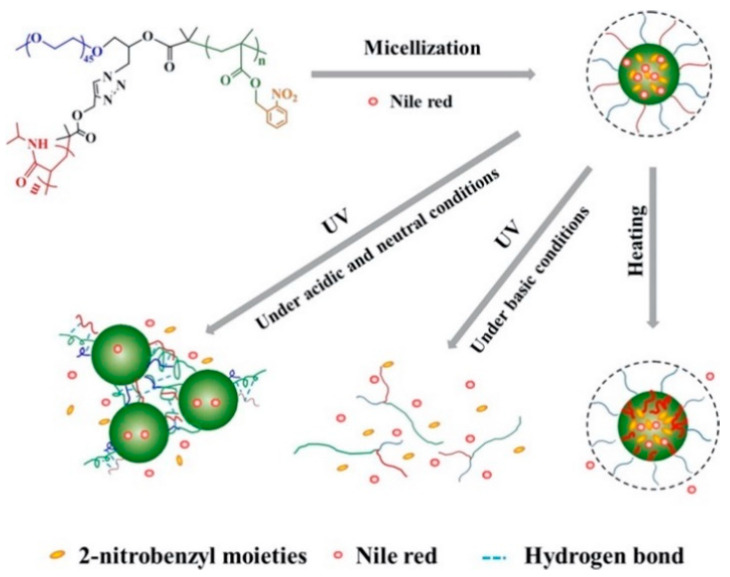
Self-assembly and dual UV/temperature response of PEG-PNBM-PNIPAM miktoarm star polymers. Reprinted with permission from reference [66]. Copyright 2017 Elsevier.

For example, the *o*-nitrobenzyl group was introduced into an ABC (A = PEG, B = poly(2-nitrobenzyl methacrylate) (PNBM), C = PNIPAM) miktoarm star, which could change its micellar morphology, in response to both light and temperature, due to its sensitive PNBM and PNIPAM arms, respectively (Figure 11) [66]. It is well known that the LCST of PNIPAM is 32 °C, but it was found to be 42 °C in the miktoarm polymer [145], which was attributed to the low MW of the PNIPAM block, as well as its clustering with PEG in the micellar coronae. Heating an aqueous micelle solution past this temperature increased micelle diameters from 97 to 142 nm, as PNIPAM chains aggregated with PNBM in the core. UV light irradiation was able to cleave and convert the *o*-nitrobenzyl functionalities into *o*-nitrobenzaldehyde and the carboxylic acid, proceeding through a Norrish II type intramolecular rearrangement. This rearrangement does not require an aqueous solvation, and as a result, can occur in micellar cores [66,146,147]. In the case of PNBM, UV-induced cleavage of *o*-nitrobenzyl moieties in its side chains converted the polymer to the now hydrophilic PMAA. DLS studies showed that the nanoparticle diameters increased by about 15 nm and had much wider dispersities. Using Nile Red as model cargo, below the LCST and without UV stimulus, its release was found to be negligible, while raising the solution temperature past the LCST only increased the cumulative release of Nile Red to 4.6% after 30 min. In contrast, UV irradiation of the sample led to a 64% release, showing the much stronger effect of converting the constituent polymer of the hydrophobic core into a hydrophilic chain.

In the photoisomerization category, azobenzenes, which are known for their ability to transition from trans to cis isomers upon UV irradiation, with an accompanying local polarity increase, are by far the most commonly employed functionalities [143,144]. This differentiates azobenzene-containing drug carriers from photocleavable systems as they can transition between their “on” and “off” states, which can allow more precise tuning of drug release at desired sites [46].

**Figure 12:**
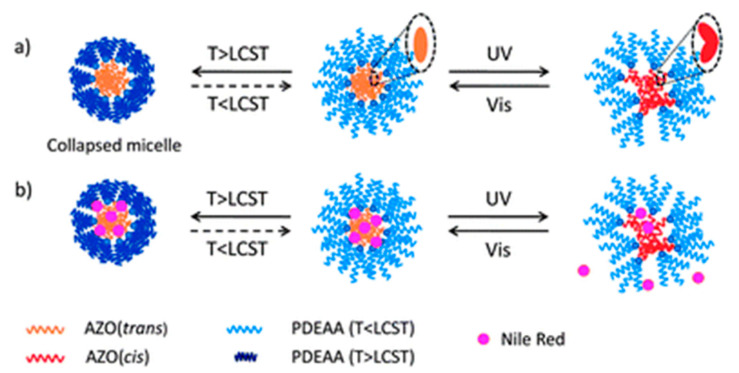
Representation of (**a**) temperature and UV-induced morphological response of PAzo-PDEAA_3_ micelles and (**b**) the corresponding proposed Nile Red release. Reprinted with permission from reference [46]. Copyright 2013 Royal Society of Chemistry.

A series of azobenzene containing AB_3_ miktoarm star polymers (A = PAzo, B = poly(*N*,*N*-diethylacrylamide) (PDEAA)) have been synthesized [46]. Using a trifunctional-azido ATRP initiator, the polymerization of an azobenzene-containing methacrylate yielded a PAzo-core structure. It was then coupled with three PDEAA arms using click chemistry to form a miktoarm polymer. The effect of temperature on the miktoarm polymer assemblies was first assessed with DSC, through which an LCST of 27 °C was determined. PDEAA became hydrophobic above this temperature and resulted in a collapse of the micelle. Temperature reversal led to a partial reassembly. Irradiation of the dual-responsive PAzo-PDEAA_3_ micelles also resulted in reversible morphological distortion, as well as an 8 nm increase in their D_h_. While one might assume that temperature-associated changes would lead to cargo release, Nile Red was found to be associated within the collapsed micellar structure. Micelle deformation from UV irradiation successfully promoted Nile Red release (Figure 12).

Another AB_3_ (A = PAzo, B = PEG) miktoarm polymer with an azobenzene-containing 4-isobutyloxyazobenzene side chain on the polymethacrylate arm has been synthesized, using a three-armed PEG macroinitiator for ATRP of the PAzo arm [53]. It formed polymersomes with diameters reported to be approximately 640 nm on average, about double that of the previously reported values. UV-vis spectra showed two absorption bands: The azobenzene *trans π*-π* transition centered at 360 nm, and the *cis* n-π* transition at 450 nm. Irradiation with a mercury low-pressure UV lamp showed complete trans-cis isomerization within 5 min, and at rest, a cis-trans reversal occurred within 24 h. Irradiation caused a morphological transition of the polymersomes to a “wrinkled” state, accompanied by a 170 nm decrease in nanoparticle diameter. The permeability of the miktoarm star polymersomes after irradiation resulted in the efficient release of encapsulated hydrophobic Nile Red or hydrophilic Rhodamine B probes.

### 4.5. Polyplex Delivery

Polymeric systems have provided a viable avenue for the delivery of genes and proteins as polyionic complexes, or polyplexes [148]. Owing to the negatively charged backbone of nucleic acids and some peptides, cationic polymer segments are often used as complexing agents for stabilization and drug delivery. As miktoarm polymers contain multiple branched segments, they can be tuned such that disparate arms can be used for complexation within the same drug delivery system. In such an example, self-assembled micelles from ABC (A = PEG, B = PLL, C = PCL) miktoarm polymers, with PEG and PLL comprising the hydrophilic corona, and PCL forming the core, were prepared [45]. Whereas PEG and PCL were used to maintain the integrity of micelles by acting as corona-stabilizing hydrophilic and core-stabilizing hydrophobic segments, respectively, PLL was used for pDNA complexation. Upon self-assembly, the 31 nm micelles were capable of loading the hydrophobic drug paclitaxel, while being complexed with pDNA, enabling their codelivery. *In vitro* studies showed a burst release of paclitaxel initially, but reached 65% cumulative release within 60 h. Unfortunately, blank micelles led to significant HeLa cell death, in which 61% of cells survived 6 μg/mL polymer concentrations. Paclitaxel loading led to a further 20% reduction in cell survival. It was found that PEG-PLL-PCL miktoarm stars formed polyplexes with pDNA through electrostatic interactions at N/P (polymer amino (N)/nucleic acid phosphate (P)) ratios higher than 2. Binding affinities were found to be similar for paclitaxel loaded and unloaded micelles. Zeta potentials were measured for N/P ratios 0.5–32, and were found to change to positive values at N/P = 2. However, these continued to steadily rise afterward indicating further structural change/binding. *In vitro* cell transfection experiments on HeLa cells revealed improved transfection in paclitaxel loaded micelles, likely due to their anti-mitotic effects.

The first example of the use of core-crosslinked miktoarm stars micelles for drug/nucleic acid delivery was for micelles that contained PEG and poly(β-hydroxyethylenediamine-L-glutamate) (PHLG) arms in their coronae [61]. Core-crosslinked polymers prepared by radical polymerization are often symmetrical [149]. The inclusion of mixed arms, as in miktoarm stars, can yield more efficient drug delivery systems, as in this case, where PEG confers solubility and PHLG was used for polyplex formation. The pK_a_ of these miktoarm stars was determined to be 8.6, thus leading to protonation of PHLG terminal amino groups at physiological pH. Due to its cationic arms, the miktoarm polymer formed a complex with negatively charged siRNA. Gel electrophoresis showed that complexes were formed at N/P ratios higher than 16, at which point, bands representing unbound siRNA were no longer seen. Flow cytometry of Cy3-labeled siRNA and Alq_3_-labeled miktoarm star cores showed that most siRNA was taken up into A549 cancer cells as a complex with miktoarm stars.

Linear (AB) and AB_3_ miktoarm stars (A = PEG, B = PGA) were synthesized based on the NCA ROP of PGA from a PEG macroinitiator, in order to study the effect of polymer topology on lysosome protein complexing [67]. Having an isoelectric point at pH 11.3, lysozyme has a net positive charge at physiological pH, which allows for its complexation with polyanions, such as PGA, which is negatively charged at physiological pH. No protein complexation was seen at pH 2, where PGA miktoarm blocks contained completely protonated carboxylic acid groups. Complexation at pH 7.4 depended heavily on polymer topology, as well as the polymer/protein ratio. At ratios of >1, complete complexation was seen for the linear AB polymers, whereas a ratio higher than 2.5 was needed for AB_3_ miktoarm stars, with the same hydrophilic/hydrophobic ratio. Interestingly, lysozyme complexation in AB_3_ miktoarm stars containing B blocks, each being the same MW as the B block in the linear copolymer, showed only marginal improvement. Based on tryptophan fluorescence, it was found that no detectable protein unfolding occurred when bound to the polymers, and so, lysozyme’s structural conformation was conserved. Despite this, lower enzymatic activity was observed for bound lysozyme, with a more noticeable decrease in lytic activity at higher polymer/protein ratios. When assessed using *Micrococcus lisodeikticus*, the lysozyme showed decreased cleavage of *N*-acetylmuramic acid and *N*-acetylglucosamine in cell walls, due to obstructed access of substrates to its active site.

AB_3_ (A = PEG, B = PGA) miktoarm polymers have also been prepared for the pulmonary delivery of complexed lysozyme as a dry powder [68,76]. The miktoarm star/lysozyme nanocomplexes were synthesized at a molar charge ratio of 2.5, with mannitol, trehalose, and leucine as aerosol excipients. Fine particle fractions of up to 68% were seen for powders consisting of the polyplexes with trehalose and leucine. Trehalose was used as a bulk agent in formulating the dry powders, which aided in stabilizing the 3D protein structure of lysozyme through the formation of hydrogen bonds. Leucine functioned as a dispersion enhancer, which was essential in forming powders with spherical morphology and an average diameter of 2.5 µm, which is ideal for inhalation. The complexes were found to be well incorporated into dry powders with encapsulation efficiencies between 80 and 100%, while retaining enzymatic activity. Additionally, conjugation of vitamin B_12_, cobalamin, to PEG termini via CuAAC click coupling, allowed targeting of B_12_-receptors in epithelial cells containing the vitamin B_12_-internalization receptor (CD320), *in vitro* and *in vivo* [76]. This significantly increased the cellular internalization of miktoarm star/lysozyme complexes compared to those without conjugated B_12_. Using the Calu-3 epithelial cell model, it was further established that the topology of miktoarm star polyplexes resulted in greater lysozyme internalization compared to linear complexes, despite a slightly stronger immunogenic response. Dry powders that incorporated the polymer/protein polyplexes showed non-homogenous dispersion in mouse lungs, yet deposition data showed that delivered lysozyme was present in the lungs 14 h after administration, in contrast to the B_12_-lacking non-targeting polyplexes.

**Figure 13:**
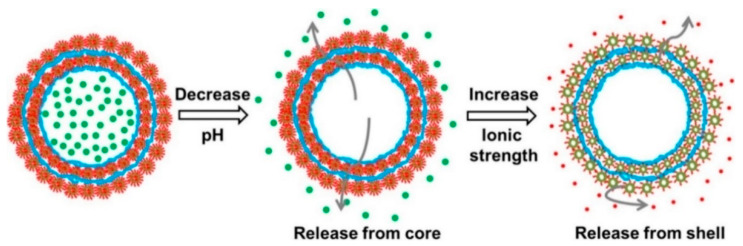
Independent release of anionic FITC-dextran and cationic rhodamine B from PEG-qPDMAEMA_4_ micelle/tannic acid-derived microcapsule cores and shells. Reprinted with permission from reference [64]. Copyright 2014 American Chemical Society.

Although not a strict example of polyplex-based delivery, a system composed of cationic bis-hydrophilic AB_4_ miktoarm stars (A = PEG, B = quaternized poly(2-(dimethylamino)ethyl methacrylate) (qPDMAEMA)) was combined with anionic poly(styrenesulfonate) to form stable polymersomes with inter-polyelectrolyte complexes [64]. Their hydrophilic interiors allowed the encapsulation of rhodamine B, a cationic dye molecule, and as a result of the polymersomes’ polyelectrolyte complexes, there was no obvious leakage of rhodamine B from their cores, but when dialyzed against a 2 M NaCl solution, the complete release was seen in 5 h. These polymersomes were used to construct multilayered microcapsules by the sequential adsorption of tannic acid and polymersome layers, on the surface of bare negatively charged silica nanoparticles, followed by silica core dissolution. PEG chains were able to strongly interact with tannic acid layers because of hydrogen bonding. A change of pH from 5 to 9 resulted in a microcapsule size change from 4.51 to 4.12 μm, which resulted in the opening of microcapsule pores (greater permeability) that could accommodate FITC-dextran in its shell at pH 5 (or below 7). Loading FITC-dextran in 8-layer microcapsule shells and subsequently switching the pH back to 9 resulted in the desorption of the dextran from the shell and its loading into the microcapsule cores. This way, both anionic FITC-dextran and cationic rhodamine B could be loaded in the microcapsule and polymersome interiors. These microcapsules could then be controlled to release either of their loaded cargoes through environmental changes in ionic strength or pH (Figure 13).

## 5. Conclusions and Future Perspective

Miktoarm star polymers are beginning to make a significant contribution to advancing the scope of drug delivery using soft polymeric nanoparticles, due to their advantageous properties, compared to the traditionally employed, and most widely studied amphiphilic block copolymers. Advances in the synthesis of miktoarm stars, using high yield methodologies, have facilitated designing formulations with better therapeutic outcomes. Methods, including arm-first and core-first, were initially explored for their syntheses, and were centered around either coupling/grafting pre-synthesized polymeric segments onto a central core, or direct polymerization from a heteromultifunctional core, respectively. These methods individually suffer from drawbacks, such as sterically induced incomplete grafting in arm-first methods, and difficulty in core initiator preparation, as well as polymerization control in core-first methods. Generally, it is best suited to combine these to tailor the overall composition of miktoarm star polymers. One of the most commonly used hydrophilic components in miktoarm stars is poly(ethylene glycol), which is commercially available in varied molecular weights, can be easily functionalized at its ends, and can be covalently linked to core molecules using highly efficient stitching methodologies, including click chemistry and condensation coupling. In-out synthesis, while a viable approach to the construction of miktoarm stars, has not been largely explored in delivering active agents, due to limitations of their self-assembled structures in drug loading in the densely packed cores.

Due in large part to their unique branching architecture, which can accommodate a variety of task-specific polymer segments, miktoarm star-based self-assemblies are formed at very low CMCs, and show high loading efficiencies, with a sustained drug release. Polymer arm tunability has permitted developing micellar formulations which could target specific organelles or cell receptors, or respond to a variety of autogenous stimuli. The ease with which branching stars can be synthetically articulated continues to offer a platform for the development of multi-stimuli-responsive systems that could deliver drugs with biological cues extra- and intracellularly. It is expected that researchers will continue to design miktoarm constructs possessing polymer segments, which upon self-assembly (i) can switch between micellar cores and coronae, in response to a stimuli, and without structural collapse; (ii) form wrinkled cores, resulting from immiscible core polymer blocks, and increase the number of binding pockets for drug cargo. Considering that noticeable progress has been made in the design and synthesis of miktoarm stars, our understanding of the structure-property relationships of their aqueous self-assembly should continue to be the next focus. This will certainly enhance our efficacy in pharmaceutical interventions in high morbidity rate diseases. Another area of growth is in theranostics, where miktoarm polymers are ideally suited to make a major contribution, due to the ease with which diverse functions, diagnostics, and delivery can be easily introduced via their structural build-up.

## Figures and Tables

**Table 1:** Miktoarm star polymers in drug delivery.

Polymeric Arms ^1,2^	Architecture	Stimulus	Cargo	Encapsulation Efficiency (%)	Loading Capacity (%)	Year	Citation
PNIPAM, PUA	AB_2_	Temperature	Prednisone acetate	27.7	N/A	2006	[27]
PEG, PCL, P2VP	ABC	pH	Nile Red	N/A	N/A	2006, 2009, 2012	[28,29,30]
PMMA, PNIPAM	AB_3_	Temperature	Prednisone acetate	55	N/A	2007	[31]
PEG, PTMC	AB_2_		Indomethacin	27.3–56.6	9.1–21.4	2008	[32]
PEG, PCL	A_2_B_2_		Ibuprofen	26.8–89.7	7.3–20.3	2009	[33]
PEG, PLLA	AB_2_		Doxorubicin Hydrochloride	72	N/A	2009	[34]
PEG, PLLA, PDLA	ABC, AB_2_		Paclitaxel	N/A	5.0–11.6	2009	[35]
PCL, PEG	A_14_B_7_		Ibuprofen	7.7–46.0	2.3–13.8	2010	[36]
PEG, PCL	A_2_B		Nimodipine	23–70	2.3–7.0	2010	[24]
PEG, PCL	AB_10_		Prednisone acetate	21.1–44.5	2.1–4.3	2010	[37]
PNIPAM, PLL	AB_2_		Prednisone acetate	18.6–21.4	2.1–2.5	2010	[38]
PEG, PS, PCL	ABC		Disperse Red 1	N/A	0.4–2.1	2010	[39]
PEG, PCL	AB_2_, A(BA)_2_		Rhodamine B isothiocyanate-Dextran	40–57	N/A	2011	[40]
PEG, Niacin, BODIPY	A_2_B, ABC		Niacin	N/A	N/A	2011	[41]
PEG, PCL, TPPBr	ABC		Coenzyme Q10	83–85	8.3–8.5	2012	[42]
PEG, PHis	AB_2_	pH	5(6)-carboxyfluorescein	N/A	0.92–1.42 μL mg^−1^	2012, 2014	[43,44]
PCL, PEG, PLL	ABC		Paclitaxel, plasmid DNA	N/A	5.0	2013	[45]
PAzo, PDEAA	AB_3_	UV light, Temperature	Nile Red	N/A	N/A	2013	[46]
PEG, PCL	AB_2_		Curcumin	48.5–65.0	4.6–6.5	2014	[47]
PCL, PBLA, PEG	ABC		Doxorubicin	56.2	11.2	2014	[48]
PEG, PCL, TIF	ABC		Curcumin	39.2–55.9	3.9–5.6	2014	[49]
PCL, PDEAEMA-*b*-PPEGMA	A_2_(BC)_2_	pH	Doxorubicin	N/A	10	2014	[50]
PCL, PDEAEMA-*b*-PPEGMA	A_3_(BC)_3_	pH	Doxorubicin	29.4–71.4	9.5–19.6	2014	[51]
PEG, PGA	AB_2_	pH	Doxorubicin	N/A	16.2–18.2	2014	[52]
PAzo, PEG	AB_3_	UV light	Nile Red, Rhodamine B,	N/A	N/A	2014	[53]
PEG, PCL	AB_2_		Doxorubicin	41.6–72.7	6.1–16.1	2015	[54]
PCL, PEG	AB_3_		Curcumin	17.3–27.6	11.4–13.3	2015	[55]
PEG, PAA, PCL	ABC	pH	Naproxen	58.2–72.8	9.7–12.1	2015	[56]
PEG, PEG-*b*-PCL	A(AB)_3_ A_2_(AB)_2_ A_3_(AB)	Redox	Camptothecin	N/A	3.6–10.8	2015	[57]
PEG, PMMA	AB_2_	Redox	Methotrexate	64	16	2015	[58]
PCL, PCL-*b*-PEG	A(AB)_3_		Doxorubicin	52.8–54.6	8.8–9.1	2016	[59]
PEG, PCL	AB_2_		Doxorubicin	24.7	5.6	2016	[60]
PEG, PHLG	A_16_B_23_ CCS		siRNA	N/A	N/A	2016	[61]
PEG, P(MMA-*co*-MAA)	AB_2_	pH	Methotrexate	48.7–82.3	10.3–16.5	2016	[62]
PCL, P(MAA-*co*-MMA)	A_2_B_6_	pH	Doxorubicin, Camptothecin	DOX: 42–60 CPT: 6	DOX: 14–20 CPT: 2	2016	[63]
PEG, qPDMAEMA	AB_4_	pH, Ionic strength	Rhodamine B, FITC-dextran	N/A	N/A	2016	[64]
PPEGMA, PMMA, PNIPAM	(BA)(AC)_2_	Temperature	Celecoxib	N/A	8.8	2016	[65]
PEG, PNBM, PNIPAM	ABC	UV light, Temperature	Nile Red	N/A	N/A	2017	[66]
PEG, PGA	AB_3_		Lysozyme	N/A	N/A	2017, 2018	[67,68]
PVDF, PS, PEG	(AB)_2_C_2_		Nile Red	N/A	N/A	2018	[69]
PCL, POEGMA	A_2_B, A_2_B_2_, AB_3_, A_3_B		Doxorubicin	34.3–62.9	4.4–8.1	2018	[70]
PEG, PCL	AB_2_		Curcumin, Methotrexate	CUR: 93.8–94.2 MTX: 72.9–75.7	CUR: 14.1 MTX: 10.9–11.4	2018	[71]
PLLA, PNAM	AB_2_		Doxorubicin	55.6–78.7	9.4–13.6	2018	[72]
PEG, PLL	AB_2_	Redox	pDNA	N/A	N/A	2018	[73]
PEG, PCL	AB_2_	Redox	Chlorin e6, Doxorubicin	Ce6: 77.7–82.1 DOX: 16.2–29.2	Ce6: 15.5–16.4 DOX: 3.2–5.8	2018	[74]
PLGA, PEG	AB_2_		Ibuprofen	51.5–76.5	1.7–7.0	2019	[75]
PEG, PGA	AB_3_		Lysozyme	N/A	N/A	2019	[76]
OA, PEG	AB_2_	pH	Vancomycin	39.6	3.6	2020	[77]
PEG, PCL	AB_2_		Baicalein	94.3–94.7	12.4	2020	[78]

^1^ Polymer segment order corresponds to the order of letters in the Architecture column. ^2^ Abbreviations used in alphabetical order: BODIPY: Boron-dipyrromethene, OA: Oleic acid, P2VP: Poly(2-vinylpyridine), PAA: Poly(acrylic acid), PAzo: Azobenzene side chain-containing polymer, PBLA: Poly(benzyl-L-aspartate), PCL: Polycaprolactone, PDEAA: Poly(*N*,*N*-diethylacrylamide), PDEAEMA: Poly(2-(diethylamino)ethyl methacrylate, PDLA: Poly(D-lactic acid), PEG: Poly(ethylene glycol), PGA: Poly(glutamic acid), PHis: Polyhistidine, PHLG: Poly(β-hydroxyethylenediamine-L-glutamate), PLGA: Poly(lactide-co-glycolide), PLL: Poly(L-lysine), PLLA: Poly(L-lactic acid), PMAA: Poly(methacrylic acid), PMMA: Poly(methyl methacrylate), PNAM: Poly(N-acryloylmorpholine), PNIPAM: Poly(N-isopropyl acrylamide), PNBM: Poly(2-nitrobenzyl methacrylate), POEGMA: Poly(oligo(ethylene glycol) monomethyl ether methacrylate), PPEGMA: Poly(poly(ethylene glycol) methyl ether methacrylate), PS: Polystyrene, PTMC: Poly(trimethylene carbonate), PUA: Poly(undecylenic acid), PVDF: Poly(vinylidene fluoride), qPDMAEMA: quaternized Poly(2-(dimethylamino)ethyl methacrylate), TIF: Tetraiodofluorescein, TPPBr: Triphenylphosphonium Bromide.
